# Effect of Variations of Amine Content and Network Branching on Thermomechanical Properties of Epoxy Systems

**DOI:** 10.1021/acsomega.4c07413

**Published:** 2024-12-11

**Authors:** Michael Robert Kelly, Arpenik Kroyan, Ingrid Hallsteinsen, Sondre Kvalvåg Schnell, Hilde Lea Lein

**Affiliations:** Department of Materials Science and Engineering, Norwegian University of Science and Technology, NTNU, Sem Sælands vei 12, 7034 Trondheim, Norway

## Abstract

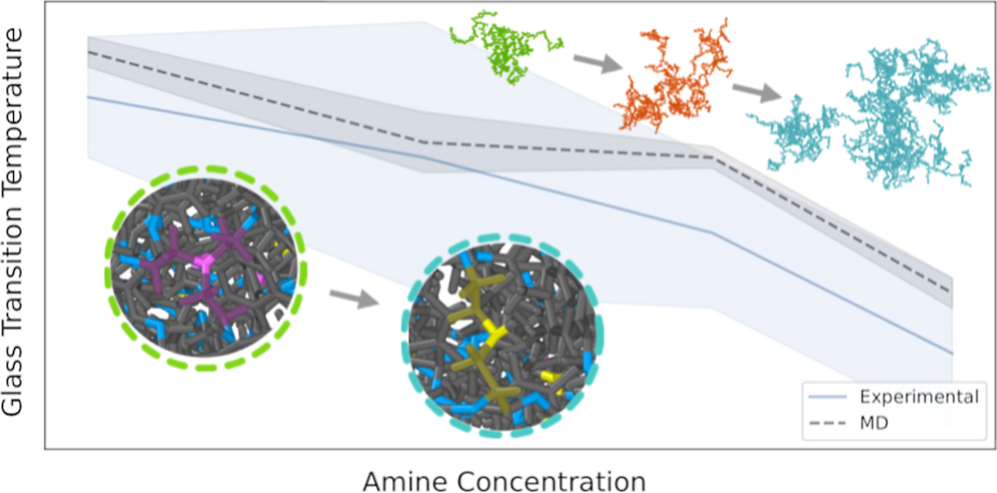

In thermosetting epoxies, thermomechanical properties can be enhanced by conscious selection of curing agents. Full cross-linking leads to a maximum in the glass- transition temperature. However, the relation between the glass transition temperature and the epoxy matrix depends on several factors beyond the cross-linking degree, such as the molecular weight of the polymers, network organization, amount of branching, and the presence of hydrogen bonds. In this study, we investigated adding non- stoichiometric ratios of the epoxy resin Epikote 828 and the curing agent Jeffamine D230. The investigations were done through a combination of molecular dynamics simulations and experiments, primarily differential scanning calorimetry and nanoindentation. Reorganization of the network to fewer clusters with a higher degree of linearity overcomes the effect of cross-linking and leads to a reduction of glass-transition temperature with increasing concentrations of curing agent to epoxy. The elastic, shear, and compressive moduli remained constant. Hence, moderating the curing agent con- tent has the potential to improve thermal properties while maintaining mechanical properties for this epoxy system.

## Introduction

The mechanical and thermal properties of the polymer matrix in epoxy-based coatings depend on the type and concentration of the curing agent used to cross-link the epoxy polymers.^1−3^ Industrially used curing agents are often based on aliphatic polyamines,^4−6^ aromatic amines,^7^ acid anhydrides,^8^ carboxylic acids,^9,10^ and Lewis base curing agents.^11^ With a variety of available substances, the desired thermomechanical properties can be crafted by careful selection of suitable curing agents, epoxy resins, and curing reaction conditions. Simultaneously, the chemical toxicity of epoxy materials generates environmental concerns and health hazards. During the curing reaction, the chemically active groups in the hardeners participate in opening the epoxide ring by creating bonds with the epoxy polymers (cross-linking). The residual amines from the curing agents are not captured in the polymer matrix in the same way, and they have been shown to have a devastating impact on human health and the environment.^12−15^ There is a growing pressure to find a ratio of curing agent to epoxy that can satisfy both the thermomechanical and environmental requirements.

Typically, an increase in curing agent results in a higher cross-linking degree in the epoxy network,^16^ where a fully cross-linked system leads to a corresponding maximum in the glass transition temperature (*T*_g_).^17−20^ Additionally, systems with higher cross-linking degrees have more rigid and compact molecular structures, which leads to a more thermally durable material,^17−20^ increased adhesion energy,^21^ higher resistance to corrosion,^22^ and higher thermal conductivity.^23^

However, the relation between the *T*_g_ and the epoxy matrix depends on several factors beyond the cross-linking degree, such as the molecular weight of the polymer chains, network organization, the presence of hydrogen bonds and fillers.^24−26^ Adding nonstoichiometric ratios of curing agent to the epoxy mixtures have also been shown to affect the *T*_g_(27,28) while the elastic modulus has been reported to be independent of the amine to epoxy stoichiometric ratio.^29−31^

**Figure 1:**
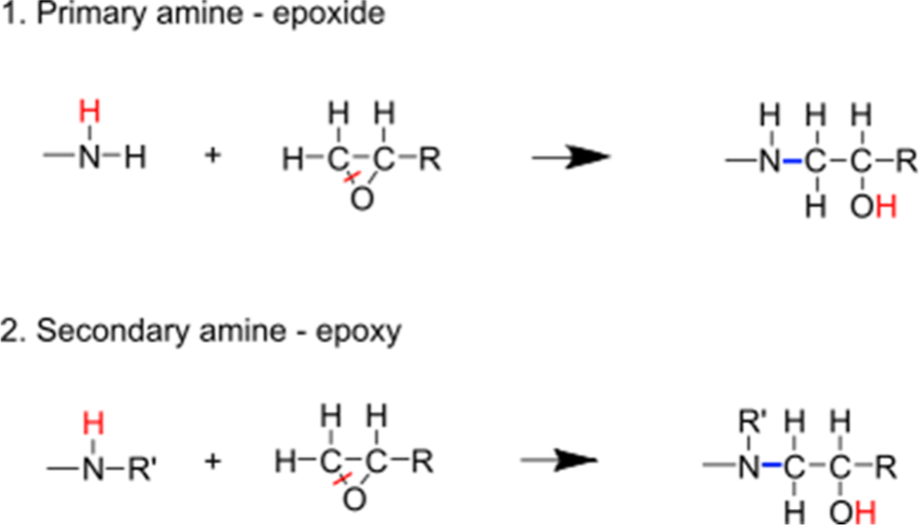
Principal reactions of epoxy curing with amine curing agents. The epoxy ring opening reaction results in a secondary amine and a hydroxyl group (1) or a tertiary amine and a hydroxyl group (2). The blue bonds are the new cross-linking bonds between the amine and the epoxide group.

The functional reactions accepted as describing the curing process for most types of amine-cured epoxy resins^32^ are shown in Figure 1.

A terminal epoxide group of the epoxy molecule opens by breaking the carbon–oxygen bond when the nitrogen from the amine group of the curing agent reacts with the carbon, creating a new bond. This bond can be formed between epoxide groups and nitrogen from primary or secondary amines. Simultaneously, the reaction generates a hydroxyl group, which can subsequently react with an epoxide group.^33^

The relationship between the cross-linking degree, the epoxy network structure, and *T*_g_ depends strongly on the type of amine and will largely determine the architecture of the resulting polymer network. Primary amines were shown in the literature to generate epoxy matrices dominated by linear segments, leading to a decrease in *T*_g_, and only a slight increase in the elastic modulus or no change in the mechanical properties of the material.^34−36^ Even though this effect has been reported previously in the literature, the fundamental reasons for such a decrease in *T*_g_ with retained mechanical properties, are not yet fully understood.

Although the bisphenol A diglycidyl ether (DGEBA) from which the Epikote 828 resin is derived is one of the more studied epoxy resins,^37^ the Epikote 828 resin is rarely mentioned in similar studies. Attempts have previously been made to combine this epoxy with a fluorinated version of the Jeffamine D230 diamine, functionalized with small amounts of fluorinated epoxy.^38^ However, the thermomechanical properties of this system were not investigated, and the molar ratio of the epoxy groups to amine groups was kept at 1:1. It is therefore interesting to consider the properties of nonstoichiometric mixtures of this epoxy system. Furthermore, DGEBA-derived epoxies were designed as a renewable alternative to aromatic fossil monomers and dominate in a wide range of industrial applications.^39^ It is therefore important to understand the hardener-dependent shifts in the thermomechanical properties of this epoxy system.

**Figure 2:**
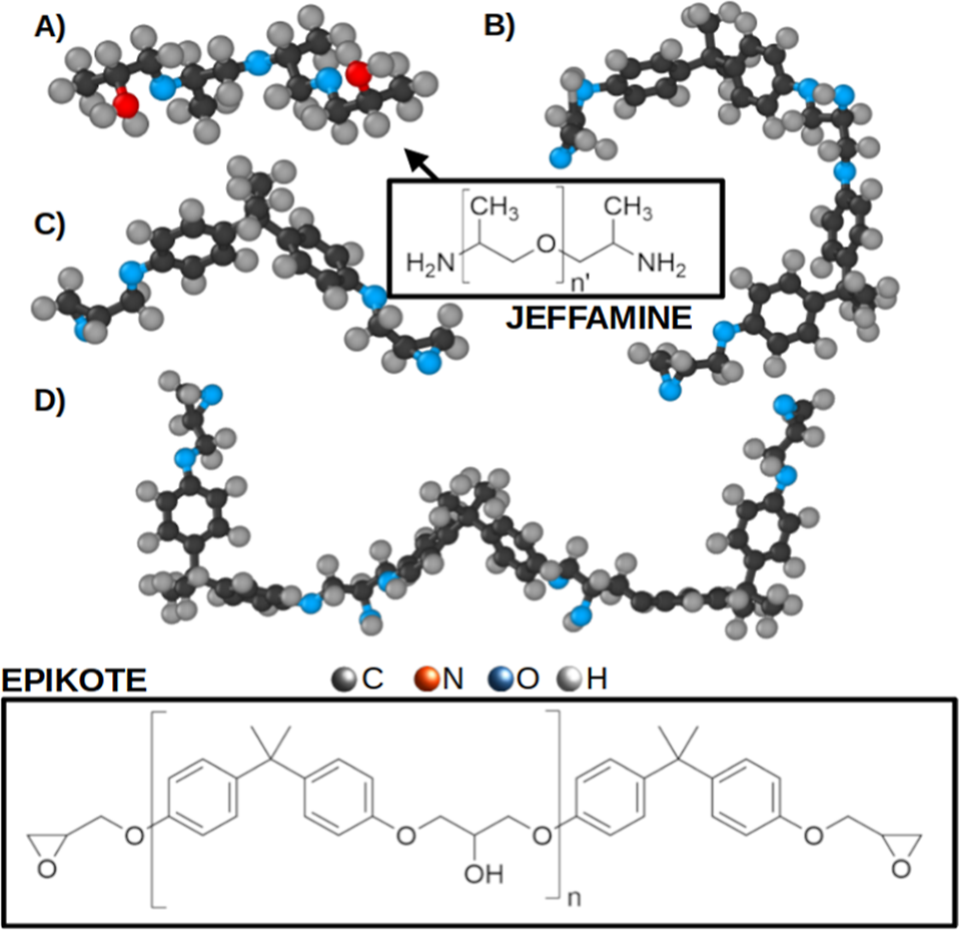
Schematic ball-and-stick representation of all-atom models of (A) poly(oxypropylene)diamine (POPDA, Jeffamine), (B) Epikote 828 *n* = 0.1, (C) bisphenol A diglycidyl ether (DGEBA) and (D) Epikote 828 *n* = 0.2. The chemical structures of Epikote resin 828 (bottom square) and Jeffamine D230 (top square), with n ranging from 0.1 to 0.2 and *n*′ ≈ 2.5. The polymers contain noninteger amounts of repeating units, denoted as n and n’, because the samples consist of a mixture of polymer chains of varying lengths. In the models used, for every eight molecules of DGEBA (where *n* = 0), there is one molecule of Epikote with one repeating unit (where *n* = 0.1) and another molecule of Epikote with two repeating units (where *n* = 0.2).

We have map the thermomechanical properties in Epikote 828 (bisphenol A-based epoxy resin^40^) using the concentration of the curing agent Jeffamine D230 (poly(propylene glycol)-bis(2-aminopropyl ether)) as a tuning parameter. Furthermore, we explain the emerging trends in the thermomechanical properties and investigate the structure–property relations in terms of changes in the epoxy network architecture. The model systems are shown in Figure 2.

The thermomechanical properties of the cured epoxies are investigated, primarily through the *T*_g_ and the reduced elastic modulus (*E*_r_). Model systems are simulated with molecular dynamics and verified against the experimentally obtained *E*_r_ and *T*_g_. The chemical structure of the networks is investigated through X-ray diffraction (XRD), both experimentally and with molecular dynamics, and through Fourier-transform infrared (FTIR) spectroscopy experimentally. Following this, the network branching architecture is studied through cluster analysis, hydrogen bond analysis, void volume analysis, and finally via the radial distribution functions (RDFs).

## Materials and Methods

### Experimental Section

The epoxy resin used was Epikote resin 828 (100%) and the curing agent used was poly(propylene glycol)-bis(2-aminopropyl ether) (100%). The epoxy resin and the curing agent were poured into glass beakers and mechanically stirred to a homogeneous mixture, and then they were put in a vacuum oven to remove air bubbles. Afterward, they were cast into greased silicon molds to obtain circular disks. This was followed by drying in the vacuum oven for 1 h.

The samples were subsequently cured at 100 °C for 5 h. Four different mixtures were made, epoxy with 20, 27, 33, and 38 wt % curing agents. These bulk samples were used for mechanical testing.

For thermal testing, the epoxy resins were dissolved in acetone (100%) in a 1:1 weight ratio mixture before the addition of the appropriate amounts of curing agent. The acetone was added to aid with the mixing of the viscous fluids. The samples were left overnight to let the acetone evaporate before curing at 60 °C for 4 h. Aluminum crucibles were used as molds.

The nanoindentation tests were carried out using a Hysitron Triboindenter TI900 with a diamond Berkovich indenter tip (Bruker Corp., Massachusetts, USA). All measurements were done at room temperature. The indentations were done in a load-controlled mode, with a loading rate of 200 μN s^–1^, from 0 to 1000 μN. The maximum load was held for 2 s before unloading at the same rate. Twenty-seven measurements were done on each sample, divided into 3 areas with 9 measurements each. These were done in a 3 × 3 grid with 50 μm spacing to avoid any potential overlap between plastic zones. The *E*_r_ was calculated using the Triboscan software (Bruker Corp., Massachusetts, USA), which uses the Oliver and Pharr method.^41^ The *E*_r_ is obtained from the slope of the load–displacement curve and is closely related to the elastic modulus (*E*) through the Poisson’s ratio (μ) as
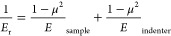
1where for the diamond indenter, *E*_indenter_ = 1140 GPa and μ_indenter_ = 0.07.^42^ The Poisson’s ratio of the sample is specific for the sample and typically varies between 0 and 0.5.^42^ For instance, for DGEBA it is 0.35.^43^ As the Poisson’s ratio of the Epikote resin 828, μ_sample_, was not found in the literature the *E*_r_, is presented in this paper.

Differential scanning calorimetry (DSC) was conducted using a Netzsch Polyma DSC 214 (NETZSCH, Bayern, Germany). The cured epoxy samples were subjected to heating and cooling cycles between 30 and 200 °C for a total of four cycles in a synthetic air atmosphere (30 mL min^–1^), while nitrogen was used as the purge gas (40 mL min^–1^). Both heating and cooling rates of 10 °C min^–1^ were employed, with cooling achieved using liquid nitrogen. Aluminum crucibles with closed lids were utilized, and an empty crucible served as the reference sample. The Proteus Analysis software (NETZSCH, Bayern, Germany) was used to determine the *T*_g_, which was identified from the temperature corresponding to the maximum of the first derivative of the heat flow curve.

FTIR spectra were collected using a Bruker Vertex 80v FTIR spectrometer (Bruker Corp., Massachusetts, USA) for both cured and uncured samples. Each spectrum was recorded in the range of 400–4000 cm^–1^, with a resolution of 4 cm^–1^ and using 128 scans.

XRD spectra were obtained using a Bruker D8 A25 DaVinci X-ray diffractometer, with Bragg–Brentano geometry and Cu Kα radiation at 1.54 Å (Bruker Corp., Massachusetts, USA). The diffractograms were assessed using DIFFRAC.EVA software (Bruker Corp., Massachusetts, USA). Data was collected from 5 to 75°, with a step size of ≈ 0.044° per step.

### Modeling

To study the effects of branching and cross-linking of the polymer matrix on the materials’ thermomechanical properties we generated a series of simulated systems (E20, E27, E33, E38), corresponding to the experimental epoxy systems with 20, 27, 33, and 38 wt % curing agents. The initial model structures were made with LigParGen,^44−46^ using the OPLS-AA force field.^47^ The Lennard-Jones potential and the Coulombic interaction cutoff was set to 10 Å, and the long-range Coulombic interactions were included using the particle–particle particle–mesh (pppm) algorithm with an accuracy of 10^–5^.

The simulations were carried out in LAMMPS (large-scale atomic/molecular massively parallel simulator), the stable version from March 2020.^48^ First, the resin and the cross-linker were mixed in the canonical ensemble (constant *N*, *V*, and *T*) in the ratios that reproduced the experimental curing agent content (Table 1). The Nosé–Hoover thermostat was set to 300 K and the system was equilibrated for 1 ns with a time step of 1 fs. In the second step, the system was compressed for 1 ns in the isothermal–isobaric ensemble using Nosé–Hoover barostat (constant *N*, *p* and *T*), with a pressure of 10 atm, temperature of 300 K and 1 fs time step, until the simulation box reached a density of 1.013 g cm^–3^ ± 0.002. In the third step, the simulation box was equilibrated in the canonical ensemble for 1 ns with a time step of 1 fs, to achieve a relaxed and well-mixed configuration of reactants.

**Table 1:** Experimental and Simulated Material Properties for the Cured Epikote 828–Jeffamine D230 Systems with Varying Curing Agent Concentrations

property	20 wt %	27 wt %	33 wt %	38 wt %
initial molar ratio of reactive groups (−)	0.4	0.6	0.8	1.0
conversion degree (%)	50	65	75	80
*T*_g,experimental_ (K)	338 ± 4	334 ± 9	329 ± 5	321 ± 5
*T*_g,MD_ (K)	341 ± 1	335 ± 1	334 ± 1	325 ± 1
*E*_elastic,experimental_ (GPa)	3.68 ± 0.25	3.63 ± 0.02	3.83 ± 0.23	3.95 ± 0.10
*E*_elastic,MD_ (GPa)	3.96 ± 0.08	3.75 ± 0.19	3.84 ± 0.06	3.88 ± 0.02
*E*_shear,MD_ (GPa)	0.71 ± 0.01	0.65 ± 0.02	0.69 ± 0.02	0.76 ± 0.01
*E*_compressive,MD_ (GPa)	2.15 ± 0.07	2.20 ± 0.03	2.23 ± 0.10	2.10 ± 0.08
H-bond/available H (%)	8.9	8.4	11.1	9.1

The cross-linking was conducted by first mapping all the epoxy rings and amine groups in the box, and then checking if two such groups appeared within a 5 Å cutoff of each other. The amine and epoxy groups that were found in that distance were reacted by assigning new bonds, angles, dihedrals, particle types, partial charges, and molecule identifiers. Once all the available reactants within the cutoff distance were processed, the matrix was relaxed and mixed in the canonical ensemble for 1 ns with 1 fs timesteps. The cross-linking, relaxing, and mixing were repeated for three iterations, as the cross-linking degree (the density of reacted molecules compared to the initial unreacted system) did not improve by further iterations. The algorithm was adapted from our previous work.^49^ The simulations were repeated for different initial geometries three times and the average values, and the standard deviations were reported in the results.

To calculate the *T*_g_, the Langevin thermostat was set to 200 K. The simulation was conducted in an isothermal–isobaric ensemble for 300,000 steps with a time step of 1 fs, maintaining a barostat pressure of 1 atm. The density was recorded every 100 steps. At the end of the run, the temperature was increased by 10 K, and the simulation was repeated. This process continued, increasing the temperature up to 500 K. The average density at each temperature was recorded, and *T*_g_ was estimated from the intersection of the linear fits at low and elevated temperatures.

The tensile and compressive modulus were simulated by deforming the box at a rate of 10^–5^ fs^–1^ over 0.1 ns along the *x*-axis in an isothermal–isobaric ensemble at 300 K and pressure of 0 atm along the *y* and *z*-axes. The shear modulus was obtained from deformation in the *xy* direction while the rest of the parameters, such as temperature and the loading rate, were kept the same. The Mooney–Rivlin derived stress–strain relation was used to generate a stress–strain curve for the calculated data points.^50^ The elastic region up to 1% strain was used to estimate the modulus.

The densities of unreacted amines, unreacted dangling ends, as well as linear and branched junctions, were calculated by mapping, labeling and counting the nitrogen atoms in the simulated systems. We determined the percentage of each species of interest relative to the total number of nitrogen atoms. This calculation demonstrates the density of different amine types — unreacted primary, linear secondary, and branched tertiary — within the systems, depending on the amine-to-epoxy stoichiometry.

Hydrogen bond densities were calculated as an average of 5000 structures sampled over 50,000 ps trajectory. The hydrogen bond donor and acceptor groups (oxygen and hydrogen of hydroxyl groups, and nitrogen and hydrogens of amine groups) were given identifiers, and their positions were mapped in the box. The hydrogen bond is accepted if the donor- hydrogen-acceptor angle was between 120 and 180° and the bond distance from the donor to the acceptor was below 3 Å.^51^ The average values were reported as a percentile of all available hydrogens. The set of parameters, such as hydrogen bond lengths and angles, were used as described in the literature for OPLS force field.^52^

The RDFs were calculated for all atom types with OCTP plug-in for LAMMPS in the canonical ensemble for 1 ns with a time step of 1 fs at a temperature of 300 K and a pressure of 1 atm.^53^ The coordination numbers were calculated as the integrals over the RDF curves.

The void volume was calculated based on the particle insertion-inflation method.^54−56^ First, a random point in the box was chosen and checked for overlaps with any atom in the polymer matrix, given the van der Waals radii of each atom estimated from the sigma parameter of the potential. The point was rejected if it violated the overlap criteria, meaning if the energy between the probe and the closest atom was repulsive. The nonoverlapping probes were accepted, and the inflation algorithm was initiated through which the radius of the inserted particle was increased until the energy between the inserted particle and its nearest neighbor became repulsive. The method was employed for 1,000,000 random points per system and the estimated histograms of the probe particle volume were included in Supporting Information Figure S1.

Cluster analysis of epoxy structure was conducted using OVITO software.^57^ The radius of gyration tensor *R*^2^ was defined as
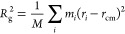
2where *M* is the total system mass, *m*_*i*_ is the individual atom mass, *r*_*i*_ is the atom position and *r*_cm_ is the center of mass position. The *R*^2^ tensor diagonal components in *x*, *y* and *z* directions were analyzed. The radius of gyration, *R*_g_ was calculated by taking the square root of the tensor, and it illustrates the span of the cluster from its center of mass.

## Results and Discussion

**Figure 3:**
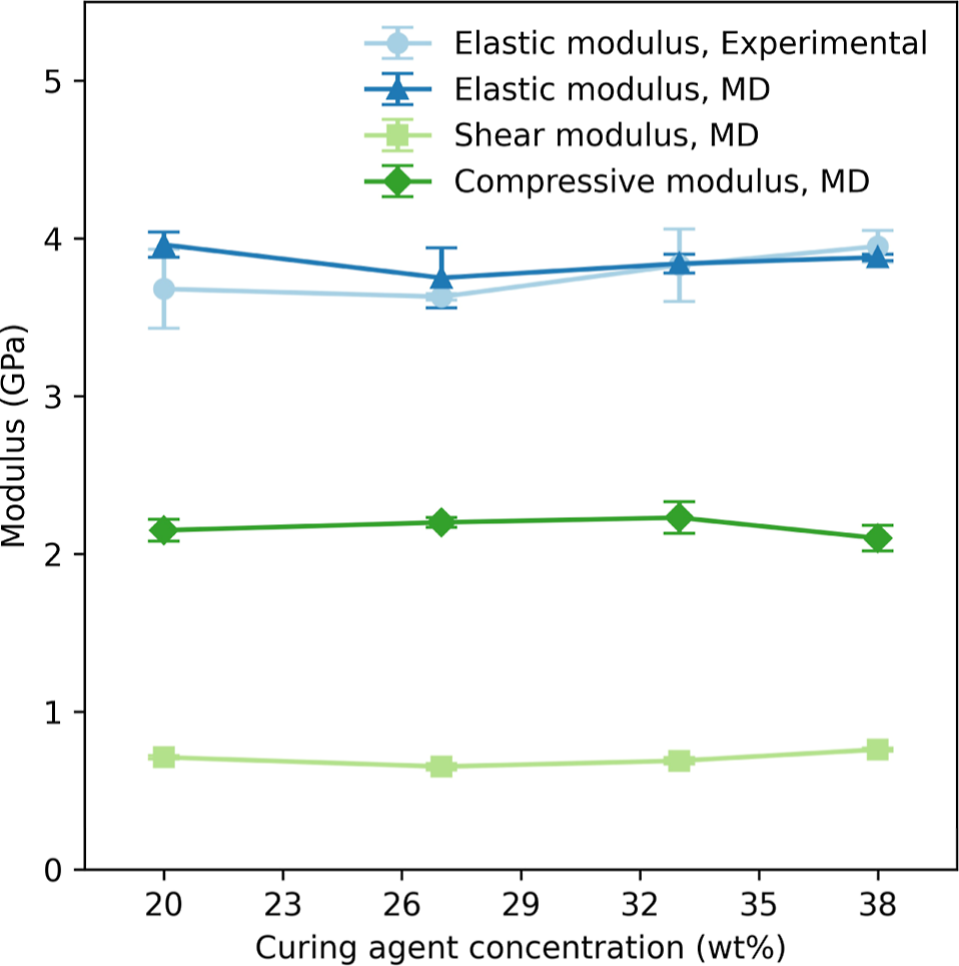
Elastic, shear and compressive modulus of the cured Epikote 828–Jeffamine D230 systems with varying curing agent concentrations from experiments (light blue) and simulations (dark). The elastic, shear and compressive modulus are largely unchanged for systems of increasing amine concentration up to the stoichiometric ratio of amine to epoxy.

The elastic modulus of the cured epoxy systems was found both experimentally and with molecular dynamics simulations. In addition, the shear modulus and the compressive mod- ulus of the systems were determined from simulations (Figure 3).

There are contrasting reports on the effect of varying curing agent concentrations on the elastic modulus in epoxy systems. Some studies report that the modulus stays constant,^29−31^ while others report a maximum at the stoichiometric amine-to-epoxy ratio.^58,59^ In the case of our systems, the epoxy network retains the mechanical properties irrespective of curing agent concentration. We found that the moduli remain nearly constant for all epoxy samples. The elastic modulus is highest, followed by the compressive modulus, and then the shear modulus. The elastic modulus was used to verify the ability of our computational model to predict the mechanical properties of this material with sufficient accuracy. The experimental and simulated results of the elastic modulus follow the same trend and agree within the standard deviations, which confirms the high quality of our model.

**Figure 4:**
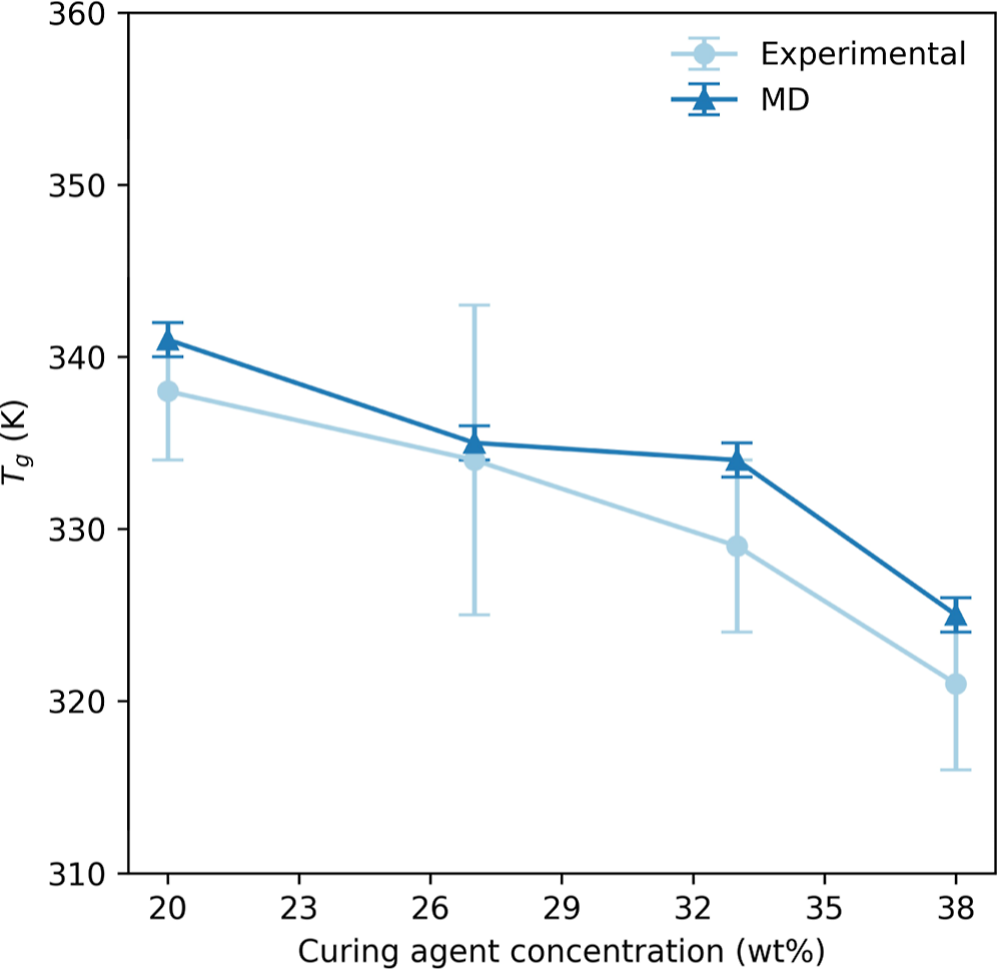
Glass transition temperatures (*T*_g_) of Epikote 828 epoxy cured with the Jeffamine D230 curing agent as a function of curing agent concentration, as acquired from experiments (light) and molecular dynamics simulations (bold).

We analyzed the thermal behavior of our material alongside its mechanical properties by measuring the *T*_g_, as shown in Figure 4.

Existing literature indicates that a specific stoichiometric ratio of amine to epoxide groups results in a maximum *T*_g_, while deviations from this ratio typically lead to a decrease in *T*_g_.^27,28^ However, our findings reveal an opposite trend: for our systems, the *T*_g_ decreases as the ratio of curing agent to epoxy approaches stoichiometry. Specifically, the *T*_g_ drop from approximately 340–325 K as the concentration of the curing agent increases from 20 to 38 wt %, resulting in a higher degree of cross-linking.

Our experimental results are consistent with the simulations, showing good agreement within one standard deviation (1 σ). This suggests that our model accurately predicts both the trends and the magnitudes of the thermal behavior of this cured epoxy.

In the absence of additives and fillers, we believe that the key factors influencing *T*_g_ are the organization of the cured epoxy network, including aspects like chain branching, void volume, and hydrogen bonding.

A similar system, utilizing DGEBA epoxy resin with the Jeffamine D230 curing agent, has been reported to exhibit a *T*_g_ of 344 to 356 K.^60,61^ These values are higher than those observed in our system. Given that DGEBA has a lower molecular weight than Epikote, we would expect it to have a lower *T*_g_. However, our systems are likely not fully cross-linked because we used nonstoichiometric amounts of the curing agent. It is anticipated that a lower conversion percentage of the epoxy groups will lead to a reduction in *T*_g_ compared to other similar systems.^62^

The presence of solvent in the system during curing can significantly affect the *T*_g_. Our findings indicate that when resin and curing agent are mixed without any solvent prior to heat treatment, the resulting cured samples exhibit a noticeable increase in *T*_g_, rising from 334 to 355 K for the 27 wt % sample, whether or not solvent is included.

Additionally, simulations applying tension to the system by compressing simulation boxes produced similar results, calculating a *T*_g_ of 357 K. This suggests that, in the absence of solvents, tensions develop in the experimental samples during curing. This phenomenon is likely due to the reduced mobility of the monomers in the mixture, which occurs when two viscous fluids, such as resin and curing agent, are mixed without solvents to facilitate mobility.

The experimental and simulated results for the moduli, *T*_g_, hydrogen bonding, and void volumes are summarized in Table 1. The number of hydrogen bonds in a cured epoxy network can significantly affect its thermomechanical properties. An increase in hydrogen bonding has been reported to enhance the moduli of epoxies.^63,64^

**Figure 5:**
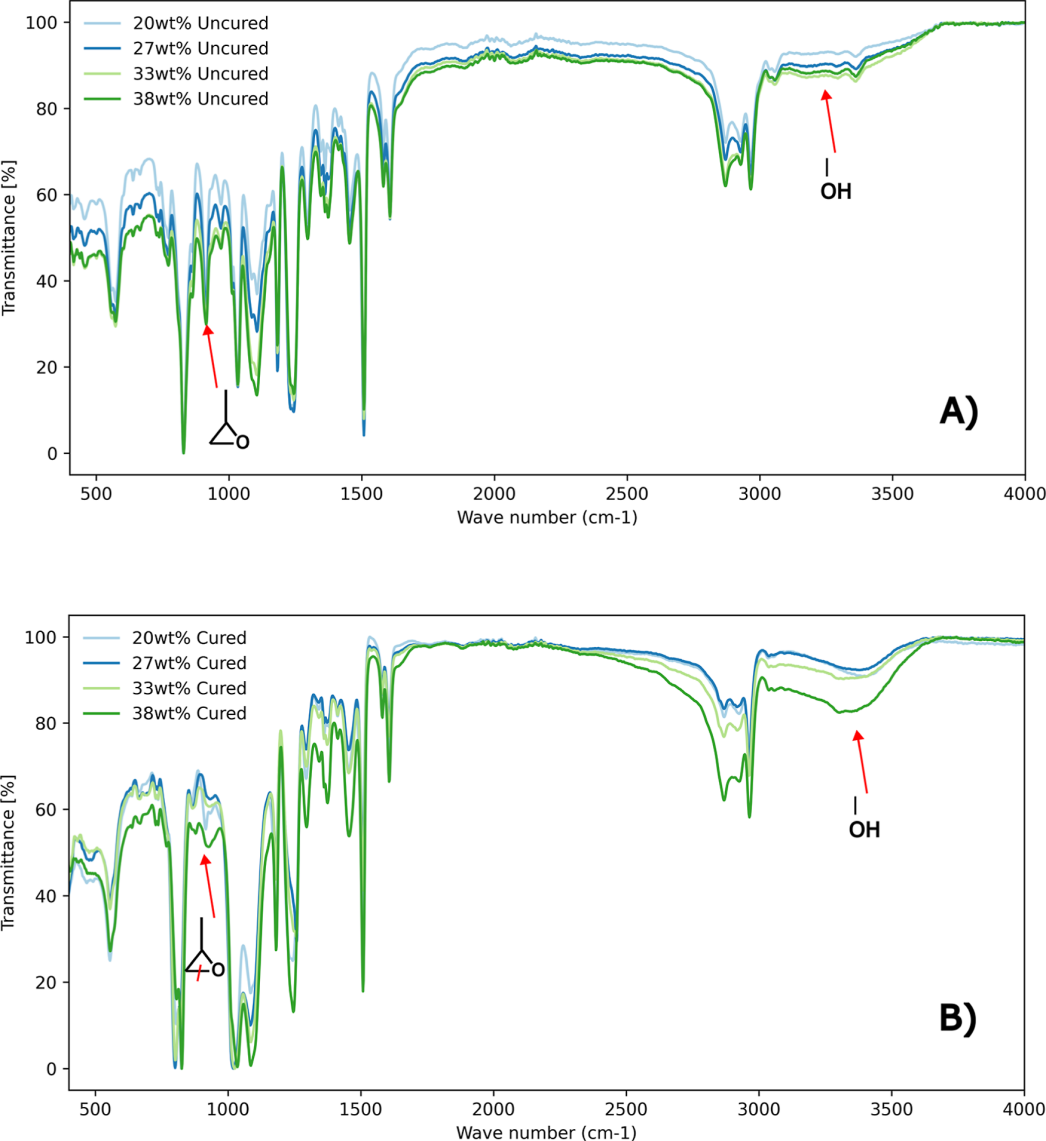
FTIR spectra of uncured (A) and cured (B) Epikote 828 with Jeffamine D230 curing agent, with varying curing agent concentrations. Arrows point to the regions of interest: the epoxide band at 915 cm^–1^ and the hydroxyl band at around 3000–3500 cm^–1^.

We mapped the hydrogen bond concentrations from simulations and found that they vary between 8% and 11% of all available hydrogen bonds (Table 1). This finding aligns with the trends observed in the mechanical and thermal properties. Additionally, we examined the FTIR spectrum of all cured epoxy systems, both before and after curing, to investigate the effect of hydrogen bonding in our epoxy networks and to confirm successful curing (Figure 5).

The spectra do not significantly differ for the different epoxy systems, the peaks generally do not shift to higher or lower wavelengths, nor do their relative intensities differ much.

However, there are noticeable differences between the spectra of the systems before (A) and after curing (B). The band at 915 cm^–1^, corresponding to the epoxide group, disappears as expected for the cured epoxy systems. The broad band at 3000–3500 cm^–1^ appears for the cured epoxy systems and is consistent with hydroxyl group stretching. The disappearance of the epoxide group and the increase of the hydroxyl band are consistent with and expected from the products of the epoxy-curing reaction.

**Figure 6:**
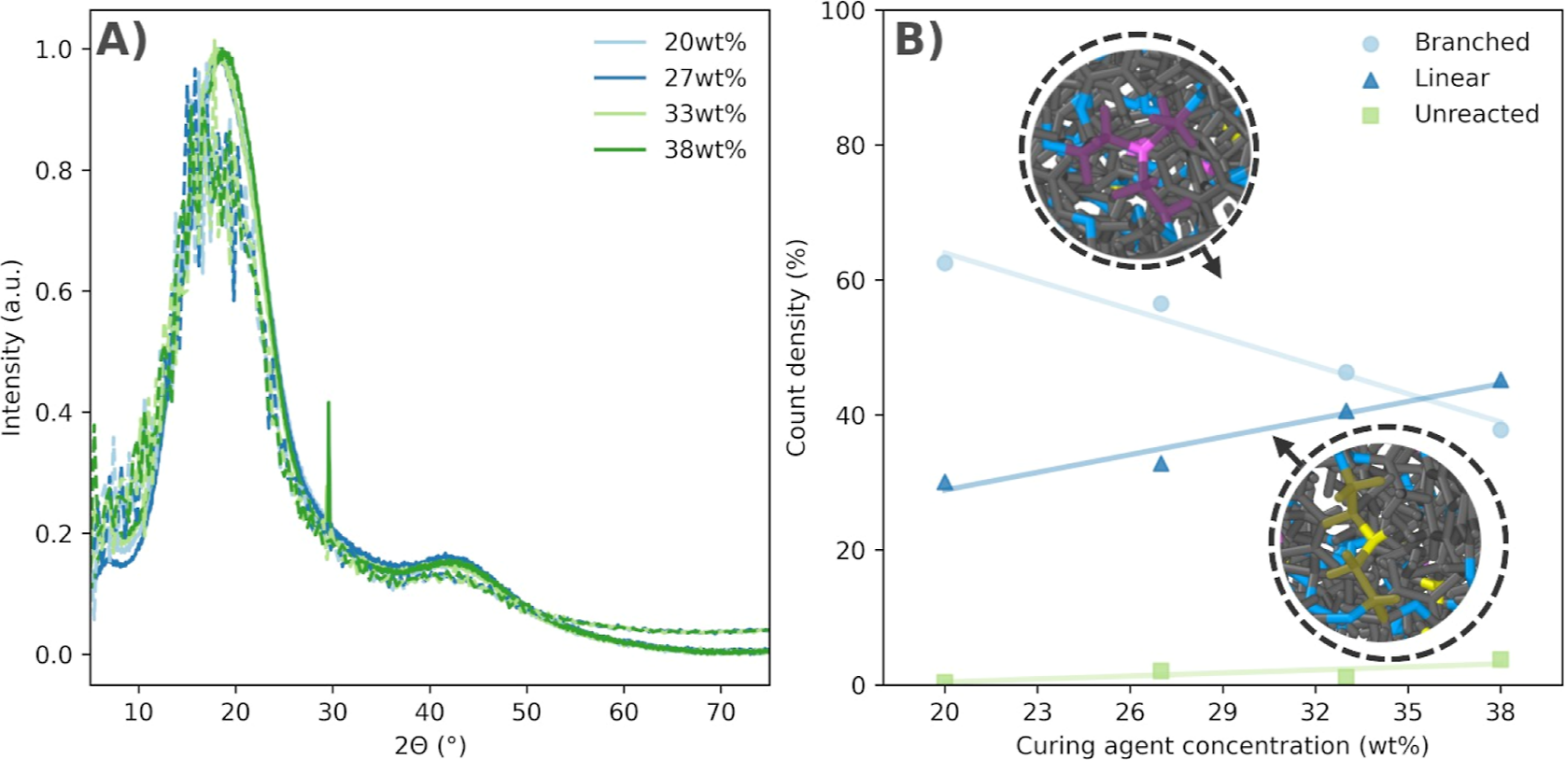
(A) XRD spectra of the cured Epikote 828–Jeffamine D230 systems with varying curing agent concentrations, as acquired from experiments (solid) and molecular dynamics simulations (dashed). The peak at ≈30° is signal from the mounting clay. (B) Count density of branched (tertiary amines) and linear (secondary amines) junctions, as well as unreacted amines (primary amines), in the Epikote 828 cured with the Jeffamine D230 curing agent as a function of curing agent concentration, as acquired from molecular dynamics simulations. Snapshots showing branched (pink) and linear (yellow) junctions are shown next to the respective data.

As discussed, varying the amounts of curing agent added to the epoxy resin before curing may influence the curing process, as the two liquids have different viscosities. This could lead to areas with significant network entanglements or semicrystallinity. However, the XRD spectra for the cured epoxy systems—both from experimental results and molecular dynamics simulations — indicate an amorphous phase in all cases, as shown in Figure 6A.

The XRD spectra of different systems exhibit similar shapes and relative amplitudes of peaks. Specifically, there are two prominent peaks: a large peak centered around 20° and a smaller peak just above 40°. Additionally, the spectra of the experimental epoxy systems contain a sharp peak at approximately 30° degrees, which is attributed to a diffraction signal from the mounting clay beneath the samples.

All samples display the same large, wide peak around 20°, which is typical for amorphous polymer networks. This indicates that varying the concentration of the curing agent does not affect the crystallinity of the epoxy systems; all samples remain amorphous with identical diffraction patterns. The polymers’ atoms are loosely held together in a disordered arrangement, lacking any long-range order.

Furthermore, the excellent agreement between experimental and simulated results rein- forces the reliability of the model predictions. Therefore, we conclude that any differences in thermomechanical properties for the Epikote systems with different curing agent concentrations are likely not due to changes in crystallinity. Instead, they may arise from variations in branching and network structure.

The amines that react with only one carbon create linear segments in the epoxy network, whereas those that react with two carbons form branched junctions. An illustration of these structures is provided in the insets of Figure 6B, based on simulation snapshots. The count density of linear and branched junctions, as well as unreacted amines, was obtained for all epoxy systems through molecular dynamics simulations, and the results are displayed in Figure 6B. A linear regression was applied to the data points to highlight emerging trends. As the concentration of the curing agent increases, the count density of branched junctions decreases, while the count of linear junctions increases simultaneously.

To further understand the relationship between linear junctions and the *T*_g_, the organization of the epoxy network was examined. One explanation in the field attributes this phenomenon to the presence of unreacted amine monomers within the polymer network. These unreacted amines can accumulate in the system as completely unreacted molecules or as partially unreacted dangling ends. The partially unreacted molecules are integrated into the epoxy network on one side of the chain while remaining unreacted on the other. Conversely, the completely unreacted amines can diffuse freely within the matrix, potentially altering the architecture of the epoxy network by increasing mobility and thus lowering the *T*_g_.^65,66^

We mapped the unreacted amine residues’ distribution in the simulation boxes (Supporting Information, Figures S5–S8), and calculated their count density (Figure 6B). The amount of unreacted amine is negligible for all systems, showing that most of the species are partially or completely reacted. It is therefore likely that the unreacted amines do not contribute significantly to the reduction of the *T*_g_ in our systems.

**Figure 7:**
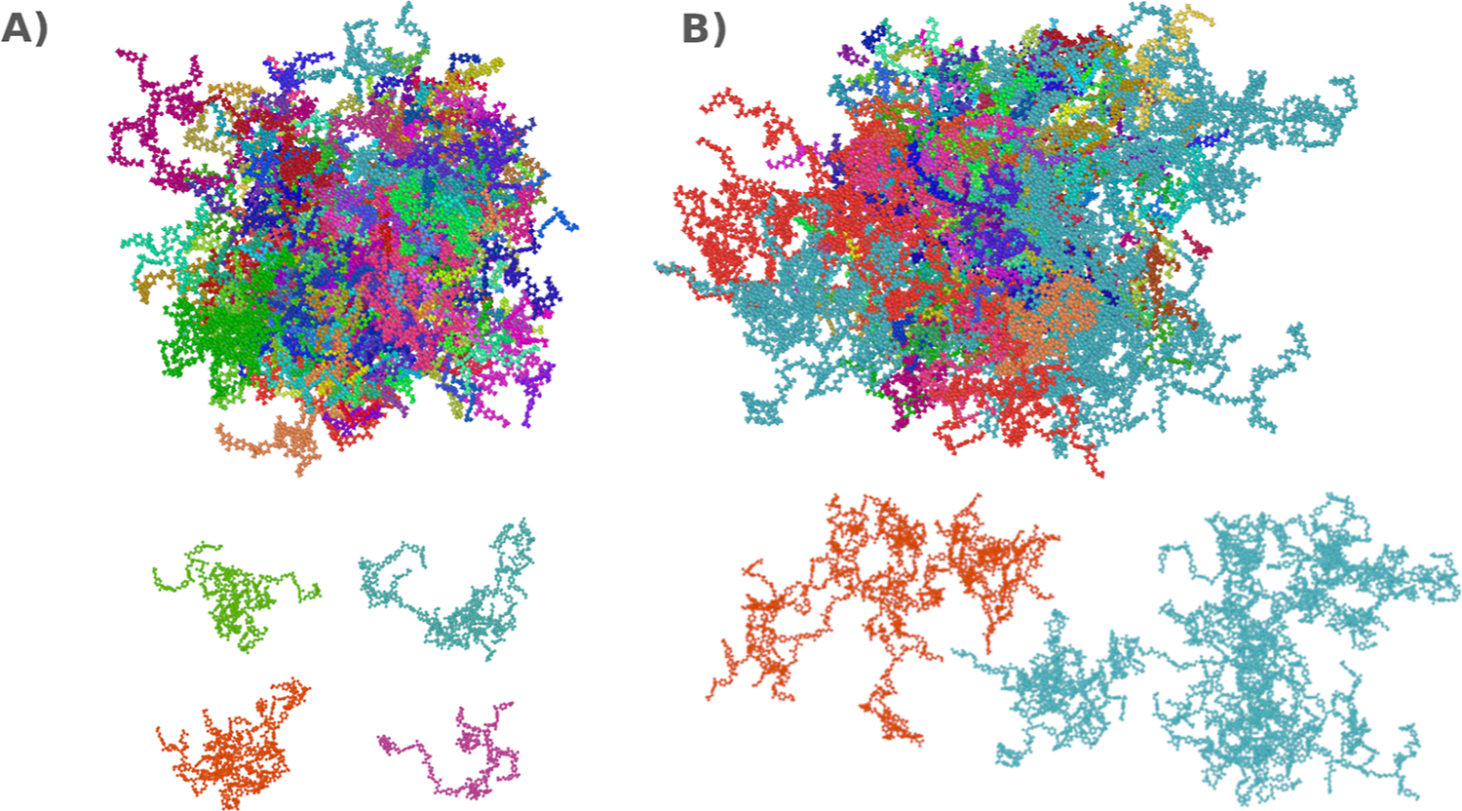
Snapshots of cluster analysis with unwrapped atoms coordinates. Epoxy networks differ substantially between the systems with (A) 20 wt % (E20) and B) 38 wt % (E38) curing agent concentrations. In lower concentration of amine the resulting structures are evenly sized, highly branched domains. However, in the case of higher amine concentration the clusters are elongated with small branched domains connected by linear segments.

Interestingly, we observe a transition from branched to linear structures as the concentration of the curing agent increases (see Figure 6B). This transition may indicate a change in the organization of the molecular clusters formed during the cross-linking reactions.^67^ We analyzed the count and distribution of these clusters in the simulation cells, with our findings presented in Figure 7.

Several key observations emerged from the results. First, we found that the average size distribution of the clusters, measured by the number of atoms, increases almost 8-fold when comparing systems with 20–38 wt % curing agent concentration. Second, the shapes of the clusters vary significantly between these systems. For the lower curing agent concentrations, the clusters exhibit evenly sized, highly branched, coil-like networks. In contrast, higher curing agent concentrations produce large, elongated networks with small domains connected by linear segments.

**Figure 8:**
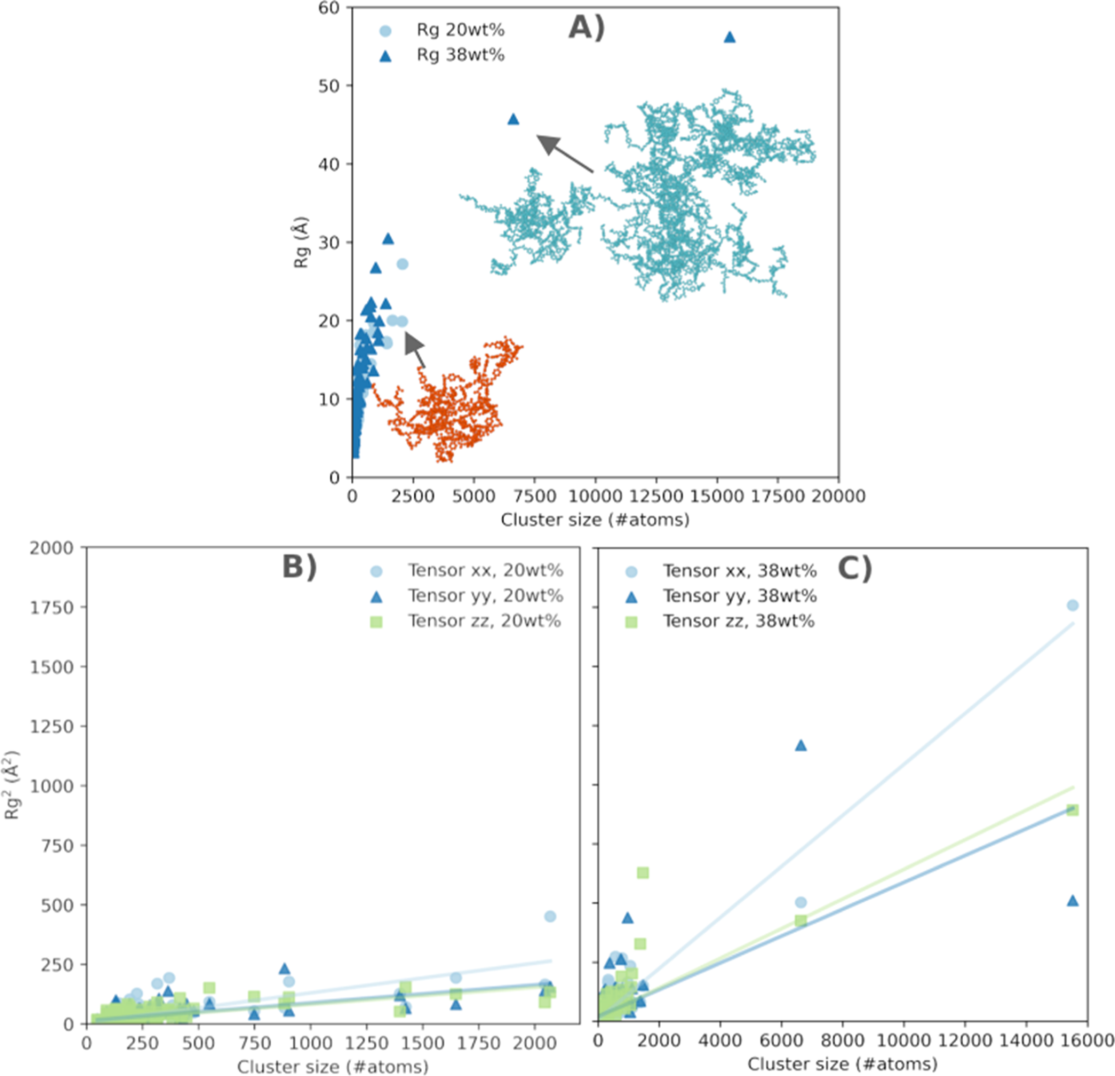
(A) The radius of gyration of the molecular clusters in E20 and E38 systems as a function of cluster size. Lower curing agent concentration leads to many small clusters while systems cured in higher amine concentration produce a few large clusters alongside the small ones. (B) The gyration tensor *R*^2^*xx*, *yy* and *zz* components as a function of the cluster size in E20 system. Most clusters are below 500 atoms large with a few stretching to 2000 atoms. For all cluster sizes their gyration tensor stretches evenly in *xx*, *yy* and *zz* directions and remains largely below 500 Å^2^. The clusters are thus approximately isotropic. (C) The gyration tensor *R*^2^*xx*, *yy* and *zz* components as a function of the cluster size in E38 g system. Most clusters are below 2000 atoms large with a few stretching to 16,000 atoms. The gyration tensor is increasing in *xx*, *yy* and *zz* directions as the cluster size increases. The clusters are slightly anisotropic in *xx* direction.

This contrasting morphology can be further characterized by the radius of gyration (eq 2, Figure 8A) of the resulting clusters, as well as the components of the gyration tensor in the *xx*, *yy*, and *zz* directions. In the 20 wt % curing agent concentration (E20), we identified 375 clusters, the majority of which contained fewer than 500 atoms, yielding a radius of gyration (*R*_g_) below 30 Å. In the 38 wt % curing agent concentration (E38), we found 194 clusters, with double the number of clusters exceeding 500 atoms compared to the E20 simulations. The largest clusters reach up to 16,000 atoms—an 8-fold increase in maximum size compared to E20—while the *R*_g_ extends up to 56 Å.

The shapes of the observed structures can be understood through the gyration tensor components (Figure 8B,C). For the low amine concentration system, the gyration tensor components in the *xx*, *yy*, and *zz* directions display evenly distributed, approximately isotropic clusters of various sizes, with values below 250 Å^2^. However, in the 38 wt % curing agent system, we see a rapid increase in the gyration tensor component values (up to 1700 Å^2^), along with larger cluster sizes and increased anisotropy in the *xx* direction.

Similarly, a transition from a multiclustered network in low amine concentrations to a single network structure at stoichiometric mixtures was documented in the literature for a tetra-functional epoxy (TGDDM) and a trifunctional epoxy (TGAP) cured with the di- amine tetra-functional 4,4′-diamino diphenyl sulfone (DDS).^67^ The authors attributed the change in network organization to mechanical properties such as higher elastic modulus, increased stiffness, and a greater degree of cross-linking for the single network structure. However, no thermal effects were investigated in that work.

In our study, we found that while the degree of cross-linking increases, the mechanical properties remain largely unchanged, and *T*_g_ decreases as the number of clusters diminishes and their size increases.

The change in the organization of the epoxy network from a multicluster to one dominated by a few large clusters might cause this appearance and the change in the distribution of microscopic “free” voids in the polymer network. Jeffrey and Pethrick^68^ reported on the relation between the network structure and the *T*_g_ and found that the stresses that accumulate in highly cross-linked networks lead to a less stable polymer matrix and a corresponding reduction in the *T*_g_. The authors argue that the vitrification of the epoxy can cause an increase in the stresses resulting from the formation of microscopic voids. The density and sizes of these voids can significantly affect the bulk density of the epoxies, and consequently, their physical properties as the chains gain space and mobility. We measured the distribution of such voids in our model systems using molecular dynamics and found only small (*r* ≤ 3 Å for cubic simulation boxes with edges 74–82 Å) spaces in the matrix with no substantial difference in the profiles between the systems with varying amine concentrations (Supporting Information, Figure S1). We estimated the total void volume in our systems, which spans from 6.4% to 5.5% and is slightly decreasing with increasing curing agent concentration and decreasing branching count. However, the results show only a slight decrease (∼1%) in this parameter and similar void distribution profiles.

**Figure 9:**
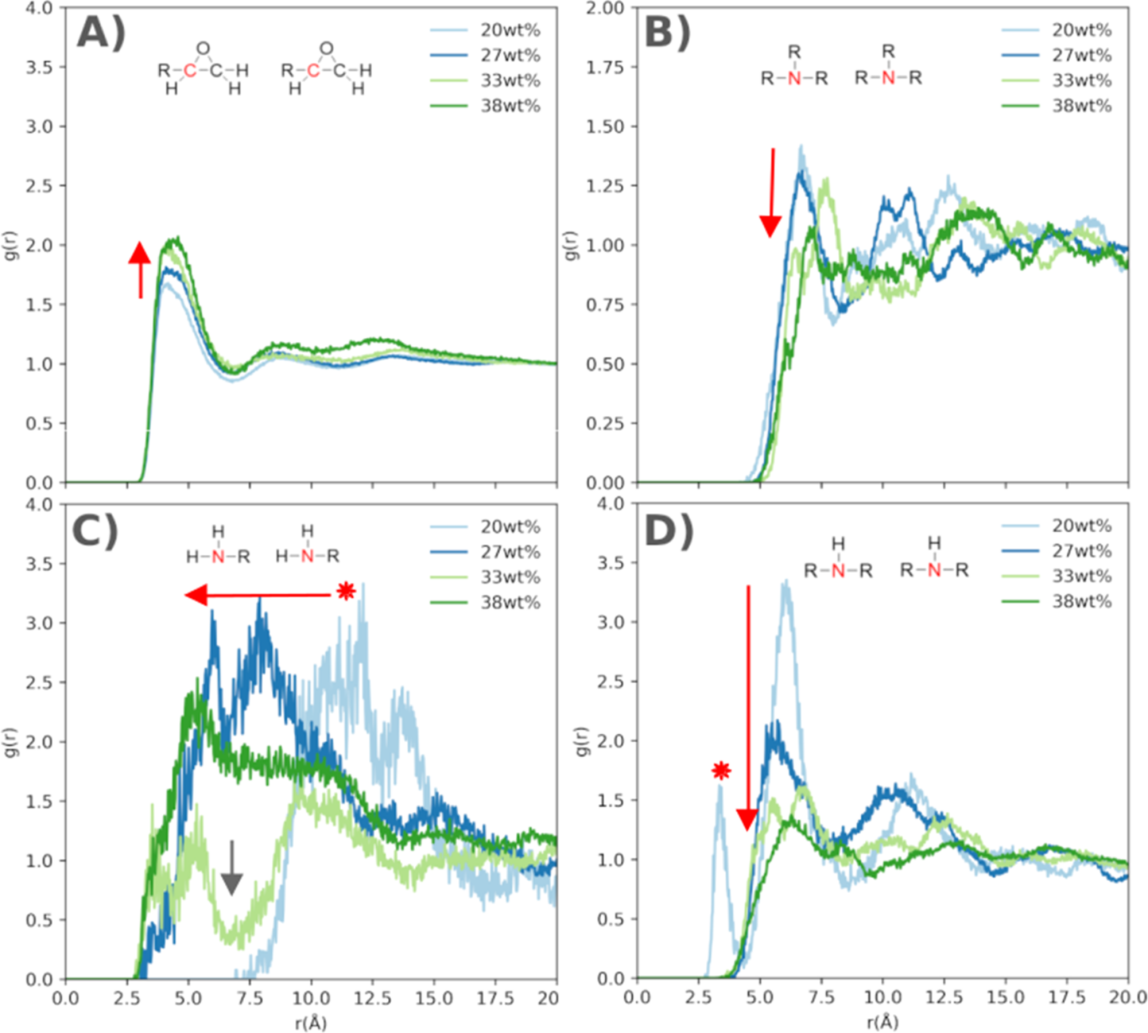
Radial distribution functions of (A) epoxide group with itself; (B) branched junctions with itself; (C) unreacted amine with itself and (D) linear junctions with itself. All RDFs presented here are from cured Epikote 828–Jeffamine D230 systems with varying curing agent concentrations. The arrows show the directions in which the correlations change with increasing curing agent concentration. The stars mark specific regions of interest.

**Figure 10:**
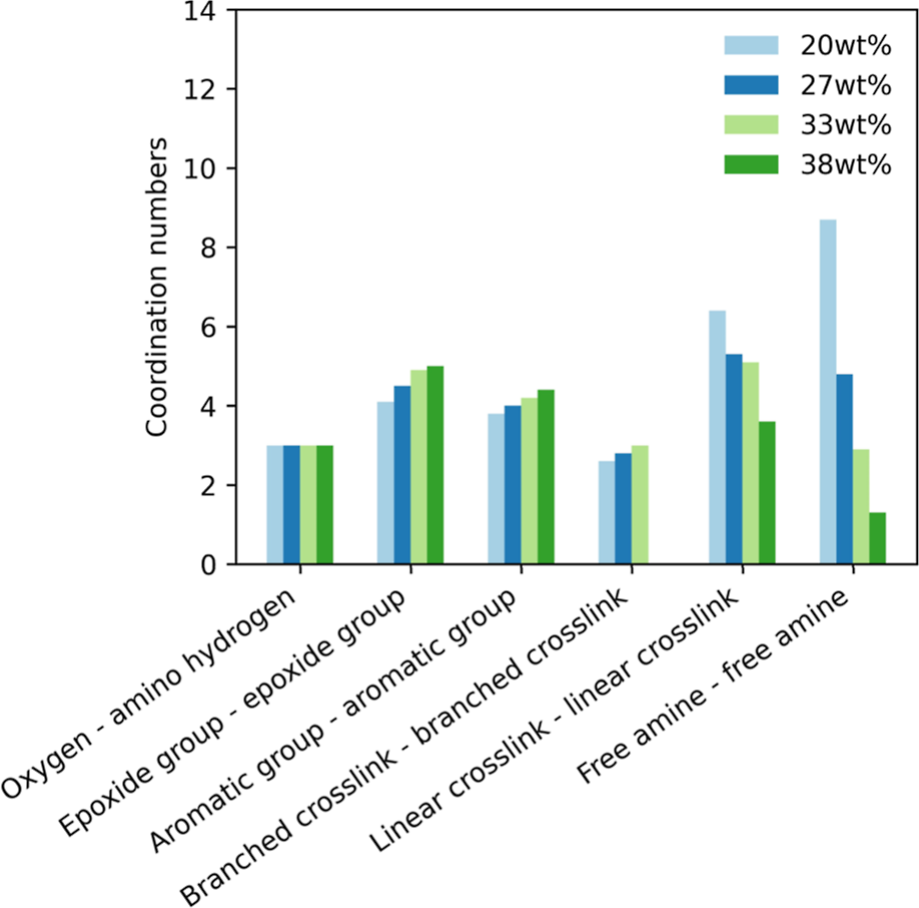
Coordination numbers for the selected radial distribution functions of the cured Epikote 828–Jeffamine D230 systems with varying curing agent concentrations, as acquired from molecular dynamics simulations.

A key step in understanding this epoxy network architecture lies in the intermolecular organization of the polymeric chains. How epoxy rings, branching junctions, linear junctions, and unreacted amines behave in the network can be captured by looking at correlations and coordinations of those specific chemical groups in the epoxy network. We investigated these patterns in the epoxy network using RDFs (Figure 9) and the coordination numbers obtained from integrating the area under the peaks of interest (Figure 10).

There are two competing effects in the cured epoxy networks. The first effect is that the interactions between epoxide rings with each other (Figure 9A) and between aromatic groups with each other (Supporting Information, Figure S4) seem to increase for systems with increased curing agent concentrations. The epoxide rings are strongly coordinated at approximately 5 Å, and the intensity of this peak rises as the degree of cross-linking increases, even though the distances do not change. This can also be seen in the coordination numbers in Figure 10; an increase in these interactions may signal an increase in the *T*_g_. Stronger interactions for systems with increased cross-linking degrees are expected to lead to a decrease in the mobility and rotation, and consequently shift the *T*_g_ to a higher value.^17−20^ The coordination between aromatic groups shows similar trends, and the nonpolar interactions seem to become heightened for the epoxy systems with higher cross-linking degrees, though these do not increase at the same rate as the interactions between epoxide groups.

As the second effect, primary, secondary, and tertiary amines become less coordinated in the epoxy networks for increasing curing agent concentrations. The RDFs of the tertiary amines with each other (Figure 9B) show the distribution of the branched junctions which are characterized by clear peaks for the samples with 20–33 wt % curing agent. The branching is still dominating in these samples. The high intensities of these peaks signal that the junctions are well organized in the matrix. For these systems, the network is structured in small, coil-like clusters. In the system with the highest curing agent concentration, the peaks are suppressed, and the branched junctions are well dispersed in the matrix. Therefore, there is no coordination number for the branched cross-links of the 38 wt % sample. We find that with higher amine concentration, the clusters change their morphology to larger, elongated domains of interconnected smaller units with linear bridges in between. The loss of highly branched clusters leads to a decrease in the *T*_g_.^17,18^

Furthermore, looking at the RDFs for the secondary amines with each other (Figure 9D), or linear cross-links with each other, similar trends are found. Initially, there is strong coordination between linear segments, and as the curing agent concentration increases, this coordination disappears. The 20 wt % sample has the highest degree of branching, so the side chains might add to the organization of the sample in the context of how the linear segments align. The small clusters are more compact; hence the linear segments are well-correlated and interlocked in the network architecture. This can be seen from three sharp peaks appearing at 3, 6 and 11 Å. The first peak (red star) disappears for higher curing agent concentrations, and the remaining peaks broaden and flatten in intensity (red arrow), which signifies a turn toward a weaker correlation. The organization and the strong interactions between the linear segments in these clusters restrict the chains and consequently decrease the mobility in the matrix, which could lead to high *T*_g_. The loss of this element in the matrix, as the cross-linking degree increases, should result in more mobile side chains, and could thereby cause a decrease in the *T*_g_. The linear cross-linking points are more uniformly dispersed in the systems with higher cross-linking degrees. In these systems, we observe a transition to large, extended clusters with smaller segments packed loosely and interconnected by long linear bridges, which allows these structures to move more freely in the network.

Lastly, the RDFs of the primary amines, both between themselves and with the unreacted amines, exhibit a consistent trend (Figure 9C). The interactions among the unreacted amines vary significantly across different epoxy systems. In systems with lower concentrations of curing agents (indicated by the red star), the amines are initially farther apart. Over time, they shift toward closer interactions (noted by the red arrow). However, these interactions remain weak, as indicated by the broad peaks.

As the concentration of the curing agent increases, we observe the emergence of a depletion region (pointed out by the gray arrow). At this stage, the unreacted amines are distributed into two regions characterized by more dispersed and weaker interactions. Finally, in the system with the highest curing agent concentration, the smoothing of the RDF indicates a lack of coordination among the unreacted amines. This finding suggests that as the curing agent concentration in the epoxy systems rises, the unreacted amines become less coordinated within the matrix. Consequently, this increased mobility may lead to a decrease in *T*_g_.

## Conclusions

The *T*_g_ of the Epikote 828 epoxy resin cured with Jeffamine D230 polyamine curing agent decreases when curing with increasing initial molar ratio of epoxy to curing agent up to the stoichiometric ratio. We found that this is due to changes in the epoxy network structure and changes in the branching hierarchy. The epoxy networks become increasingly linear for systems with higher concentrations of the curing agent. There is no change in the density of hydrogen bonds, nor any crystallinity that may affect the thermomechanical properties. The elastic, shear, and compressive moduli remain unchanged for all systems. We conclude that the decrease in the *T*_g_ as the curing agent concentration increases is due to a loss of coordination between cross-link junctions in the epoxy network, accompanied by a transformation in the cluster architecture from multicluster to one dominated by a few large clusters. Since the *T*_g_ decreases for higher initial curing agent concentrations, while the moduli remain largely unaffected, the use of less curing agents is recommended for use in coating applications, where thermal stability is desired. Nonstoichiometric mixtures of the amine curing agent and the epoxy resin can therefore give improved thermal stability while maintaining the mechanical properties. Future research may benefit from an in-depth look into the different cross-linking conditions and mixing ratios one may tune to achieve a desired network structure or thermal or mechanical performance, suited for their specific applications.

## Supporting Information Available

Additional results from molecular dynamics simulations, such as probe particle volume distributions, radial distribution functions and amine density distributions from simulation snapshots are to be found in the Supporting Information section (ZIP)
(PDF)
