# Supplementary figures and images for: Effect of Variations of Amine Content and Network Branching on Thermomechanical Properties of Epoxy Systems
Source: ACS Omega. 2024 Dec 11;9(51):50414–26. doi: 10.1021/acsomega.4c07413 (PMC11683609; doi:10.1021/acsomega.4c07413)

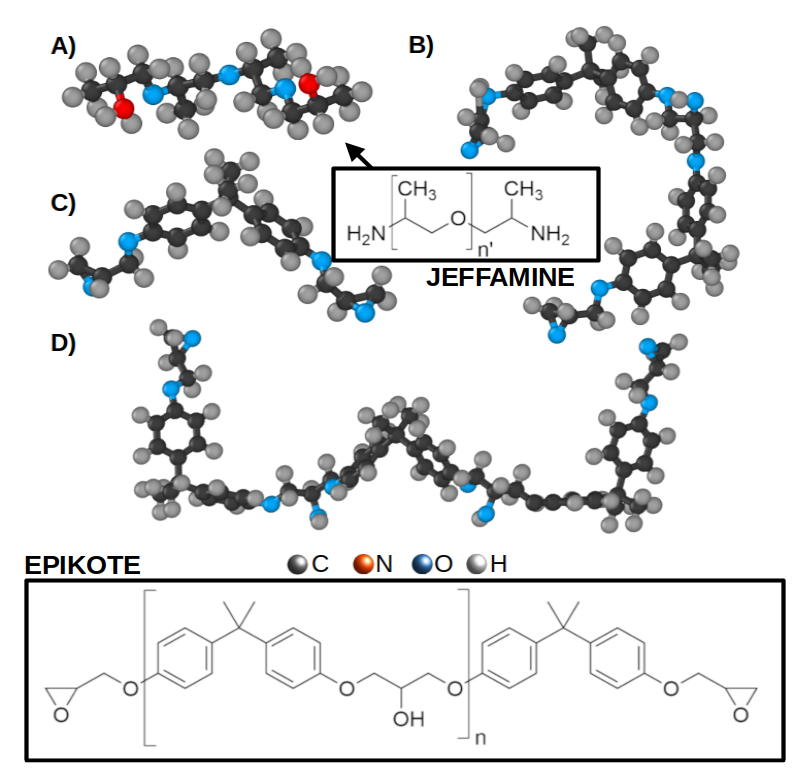

Supplement: Supplementary file 1 — ao4c07413_si_001.zip [file ao4c07413_si_001.zip › graphics/models.png]

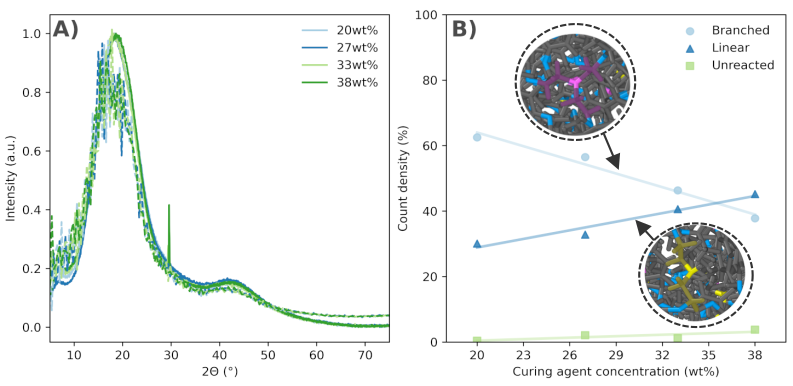

Supplement: Supplementary file 1 — ao4c07413_si_001.zip [file ao4c07413_si_001.zip › graphics/xrd_count.png]

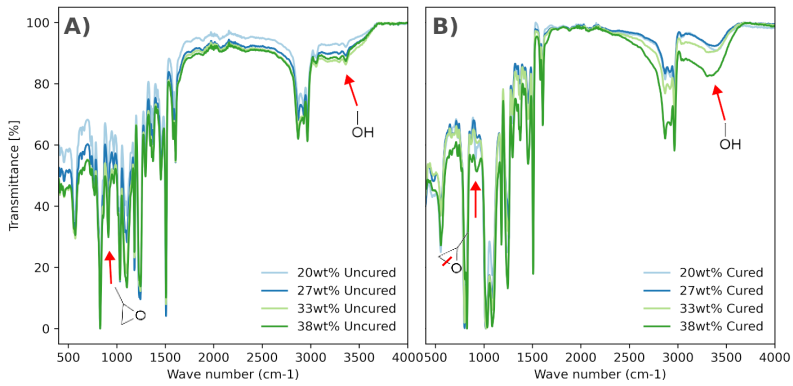

Supplement: Supplementary file 1 — ao4c07413_si_001.zip [file ao4c07413_si_001.zip › graphics/ftir.png]

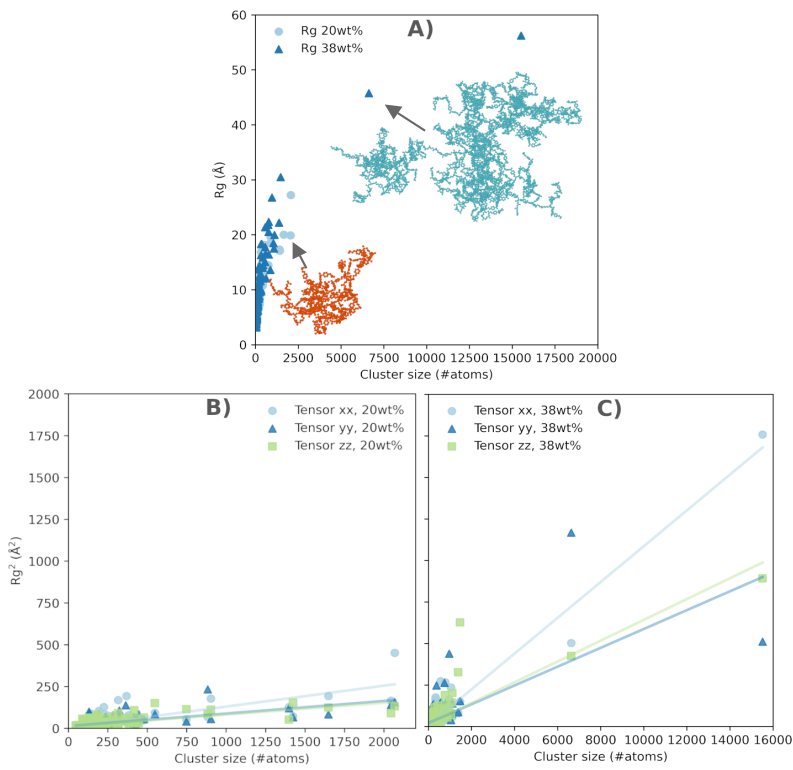

Supplement: Supplementary file 1 — ao4c07413_si_001.zip [file ao4c07413_si_001.zip › graphics/gr.png]

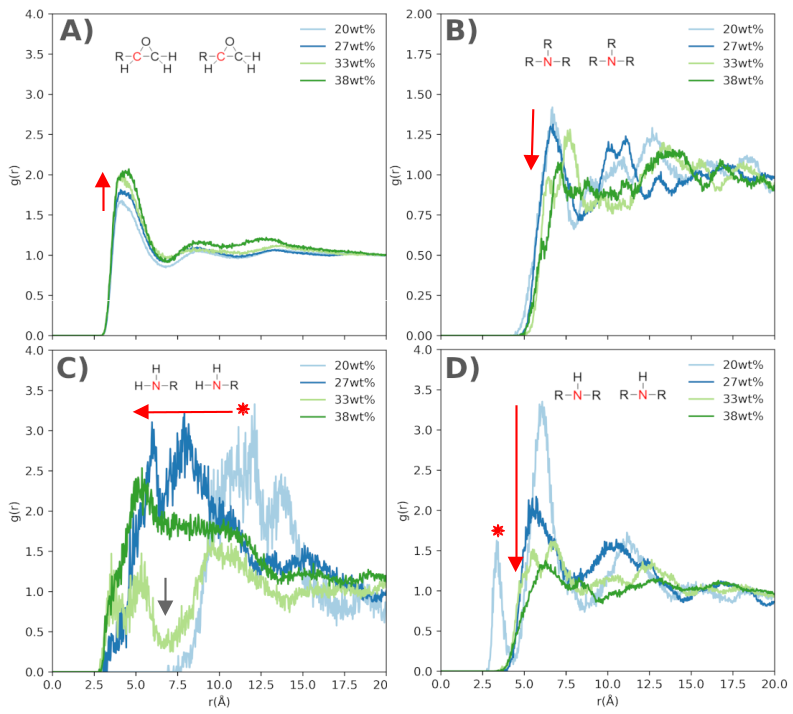

Supplement: Supplementary file 1 — ao4c07413_si_001.zip [file ao4c07413_si_001.zip › graphics/rdf_fin.png]

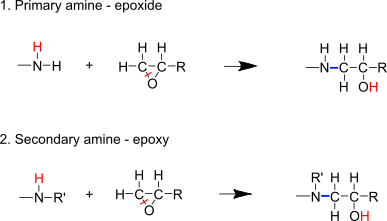

Supplement: Supplementary file 1 — ao4c07413_si_001.zip [file ao4c07413_si_001.zip › graphics/curing2 v3.png]

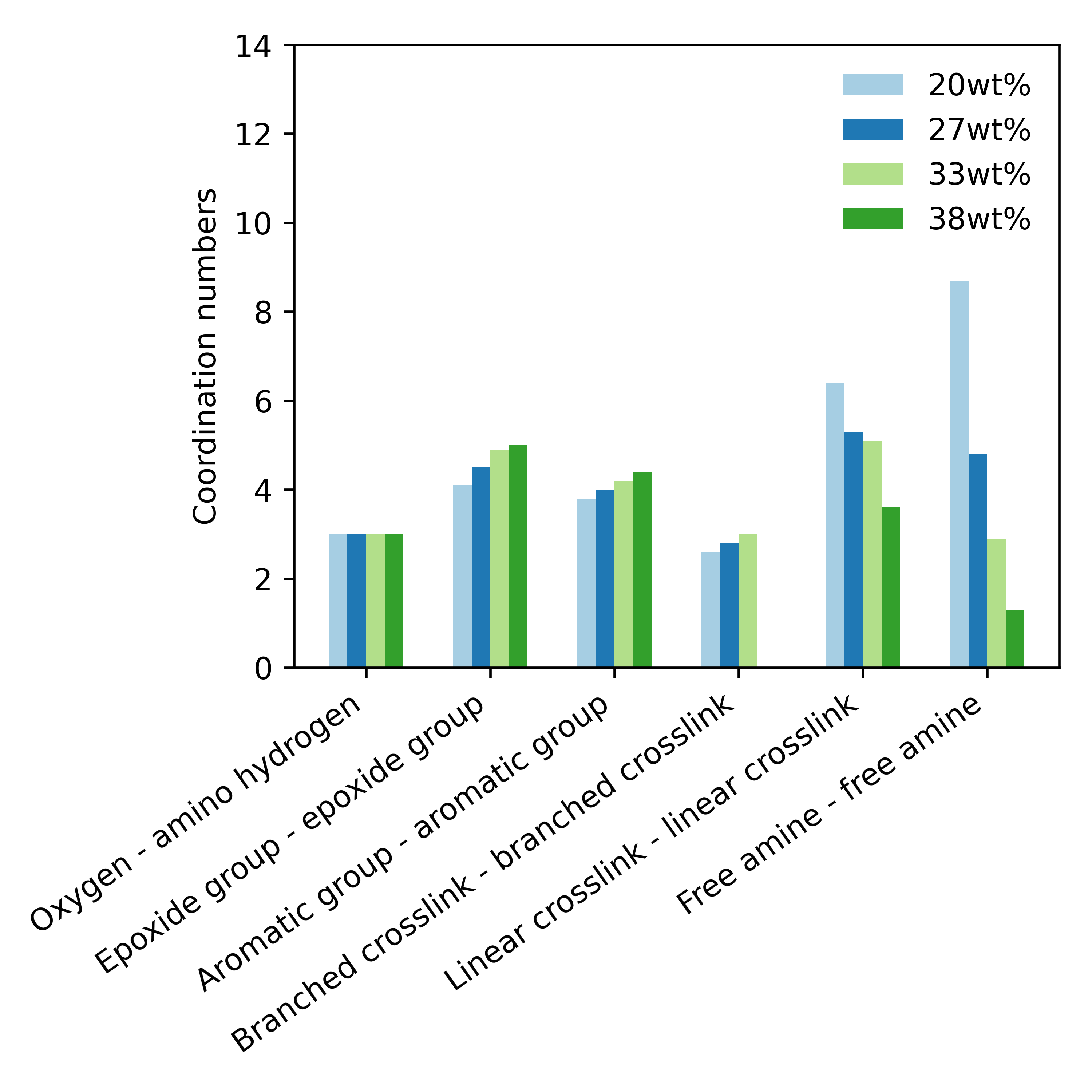

Supplement: Supplementary file 1 — ao4c07413_si_001.zip [file ao4c07413_si_001.zip › graphics/Results/Results coordination numbers.png]

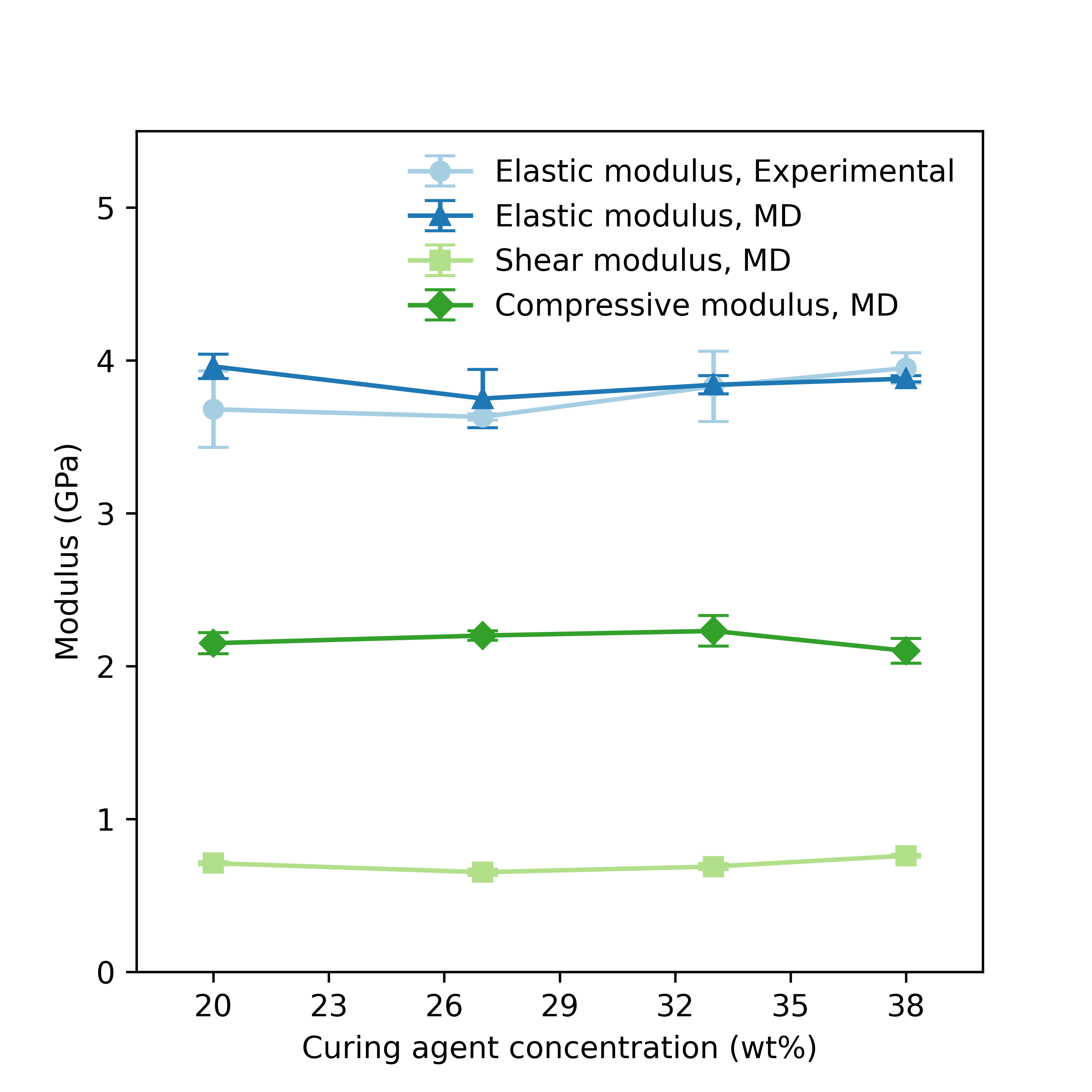

Supplement: Supplementary file 1 — ao4c07413_si_001.zip [file ao4c07413_si_001.zip › graphics/Results/Results modulus.png]

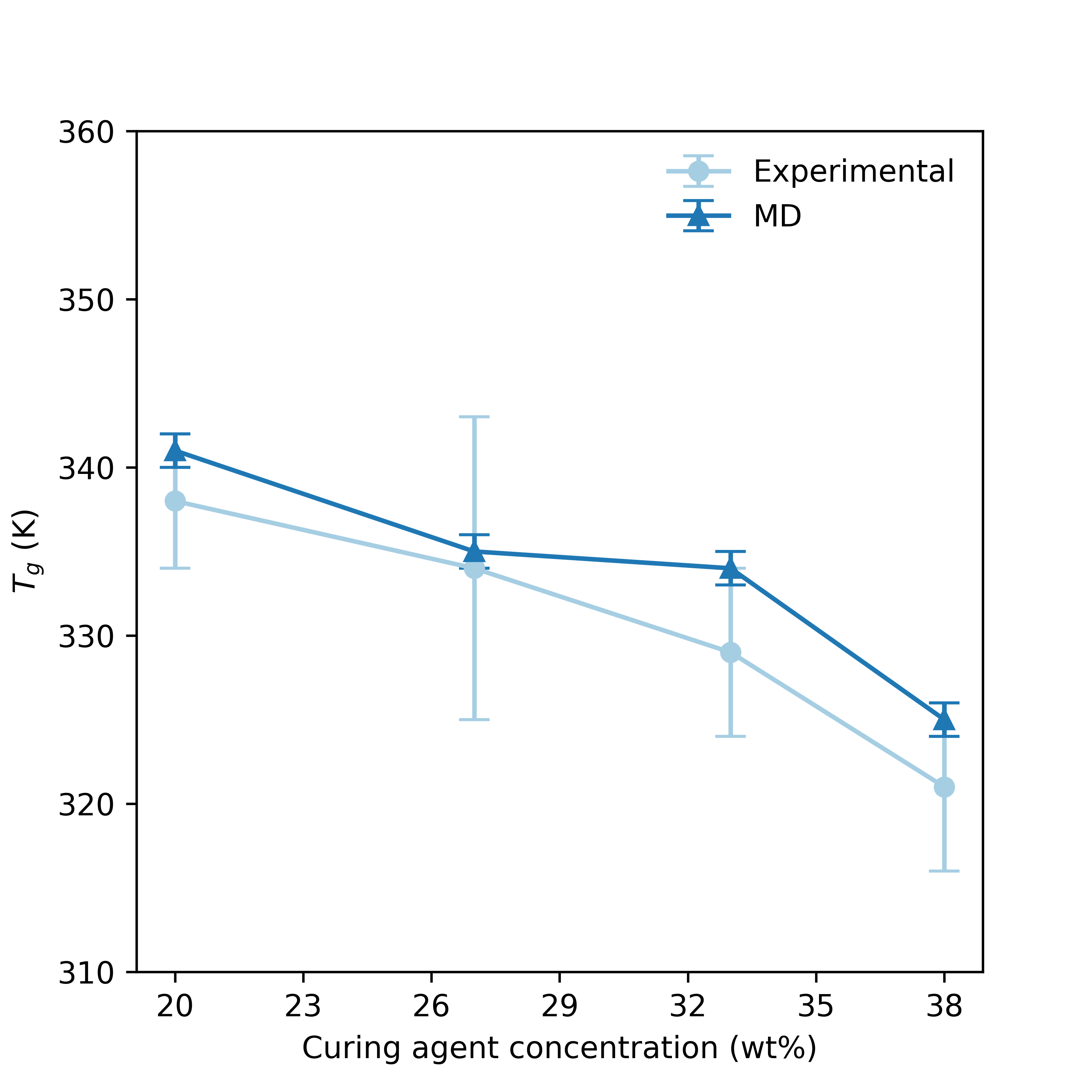

Supplement: Supplementary file 1 — ao4c07413_si_001.zip [file ao4c07413_si_001.zip › graphics/Results/Results Tg.png]

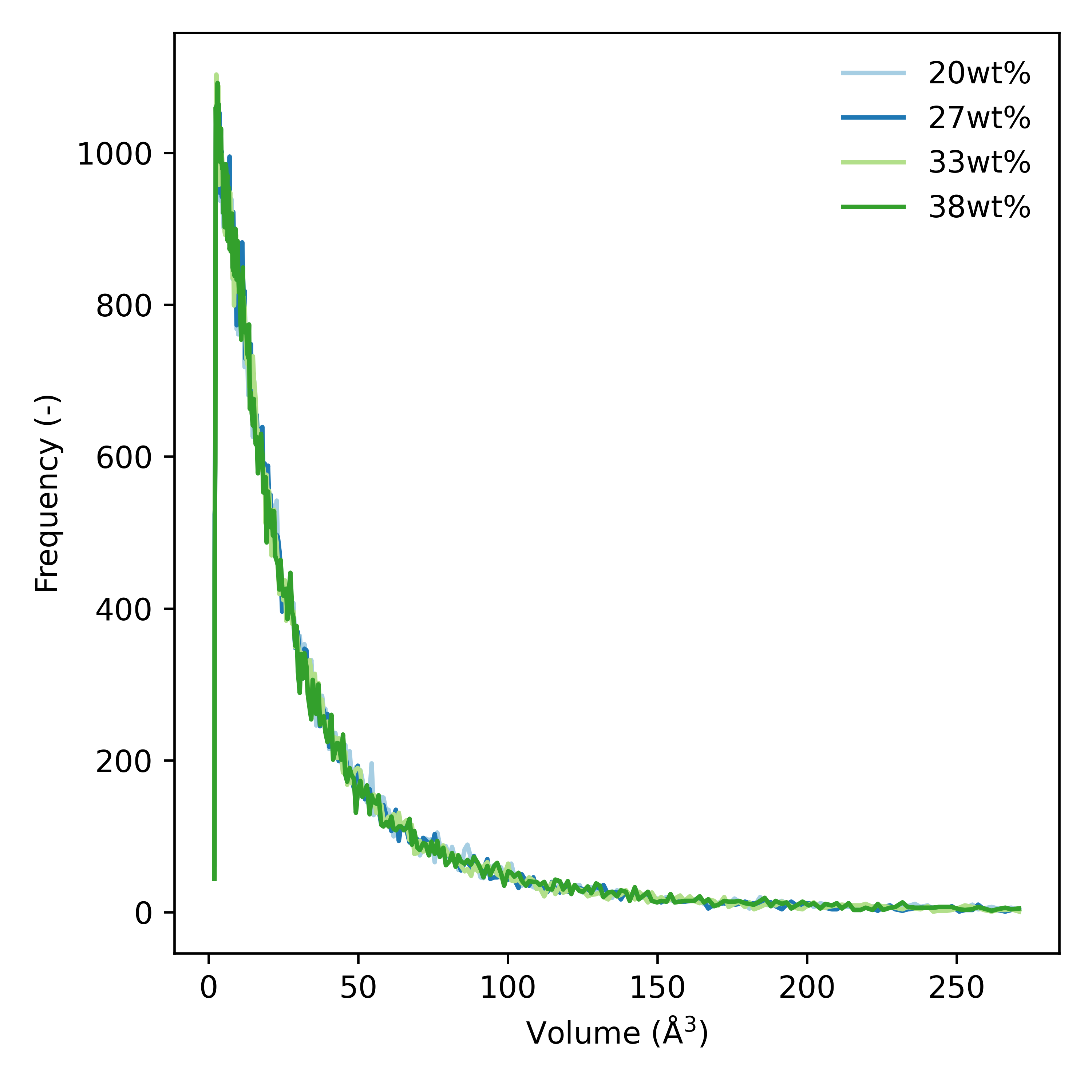

Supplement: Supplementary file 1 — ao4c07413_si_001.zip [file ao4c07413_si_001.zip › graphics/Results/Results volume.png]

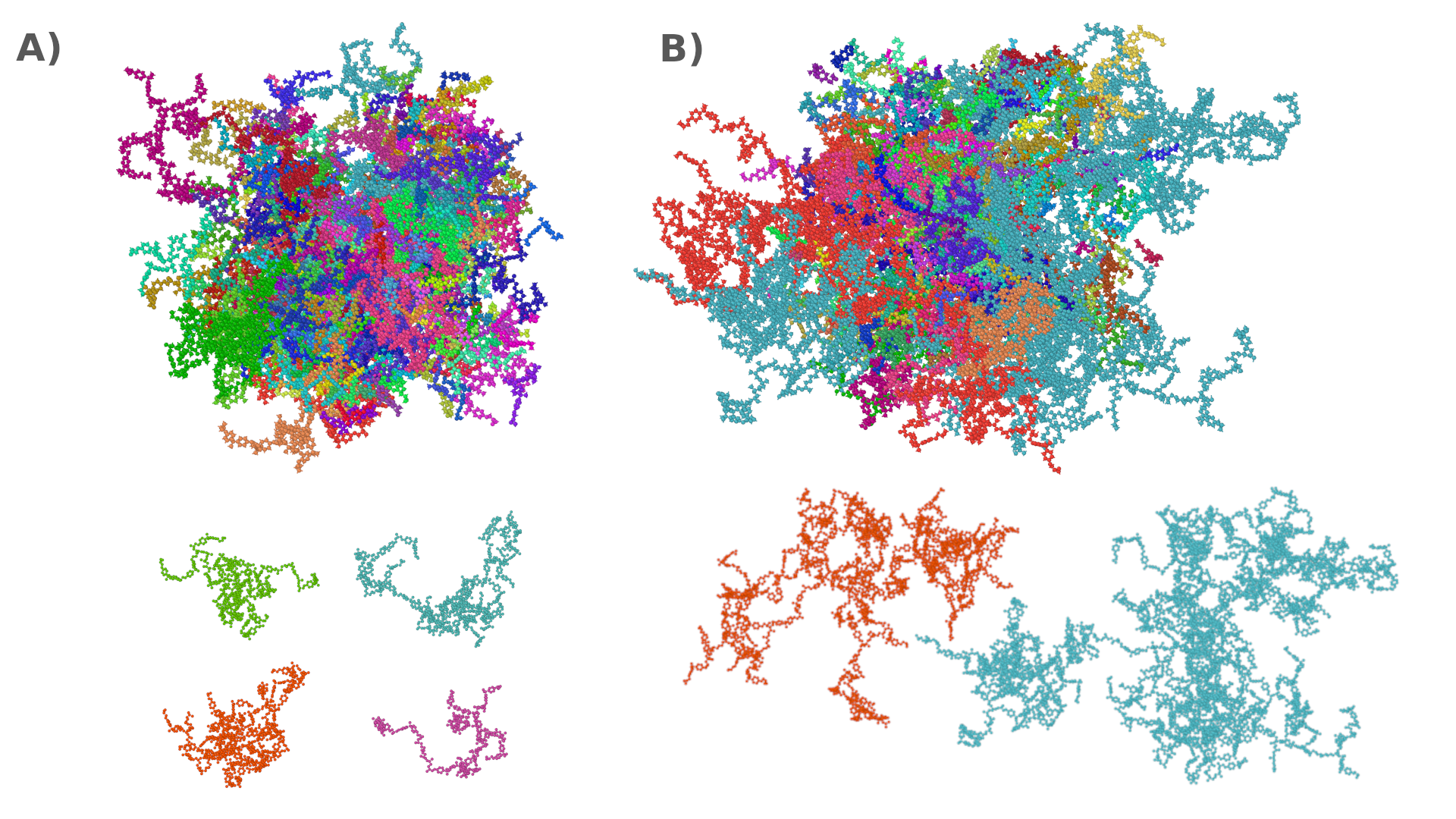

Supplement: Supplementary file 1 — ao4c07413_si_001.zip [file ao4c07413_si_001.zip › graphics/Results/Clusters.png]

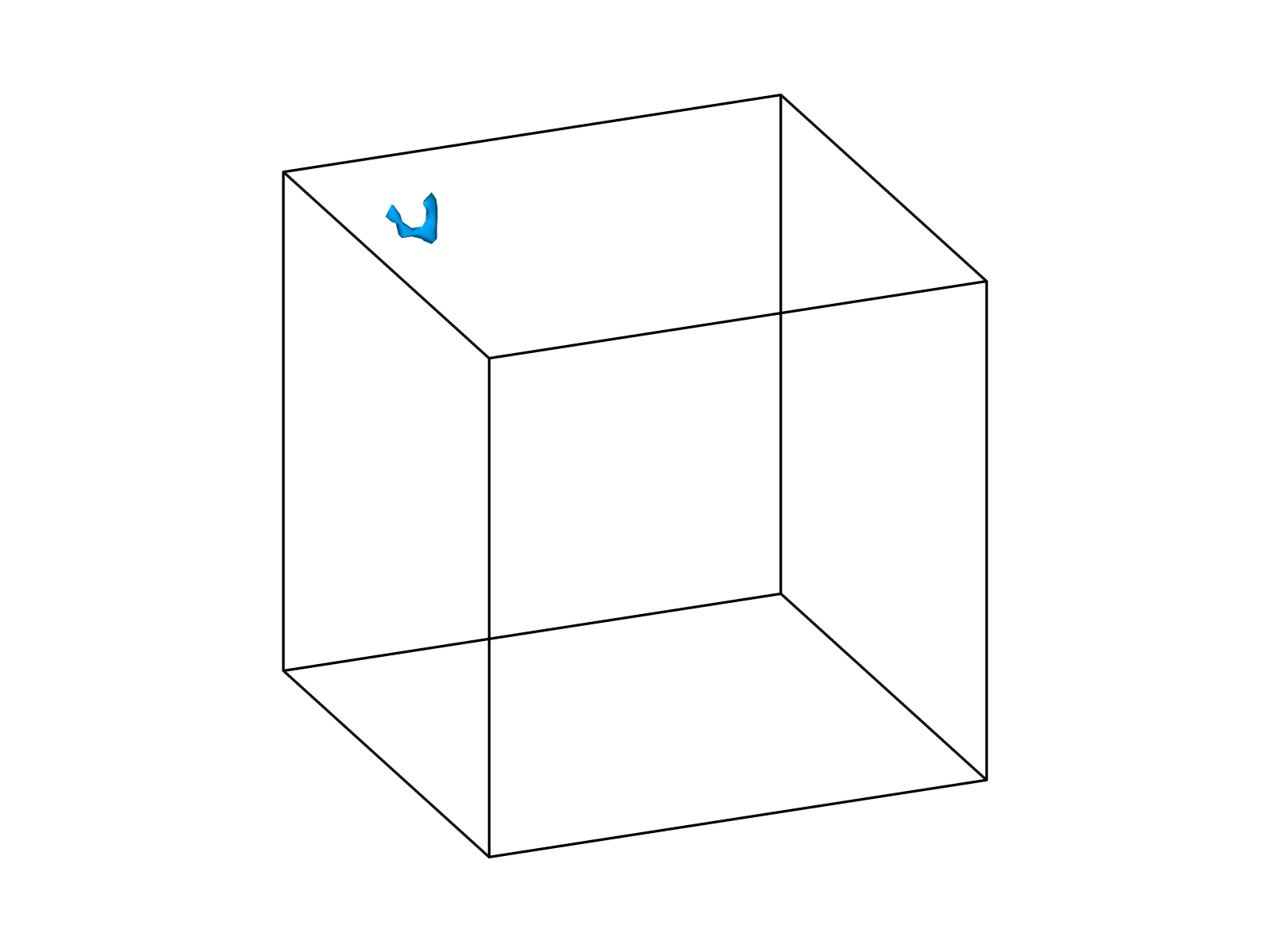

Supplement: Supplementary file 1 — ao4c07413_si_001.zip [file ao4c07413_si_001.zip › graphics/Results/amine_e25.png]

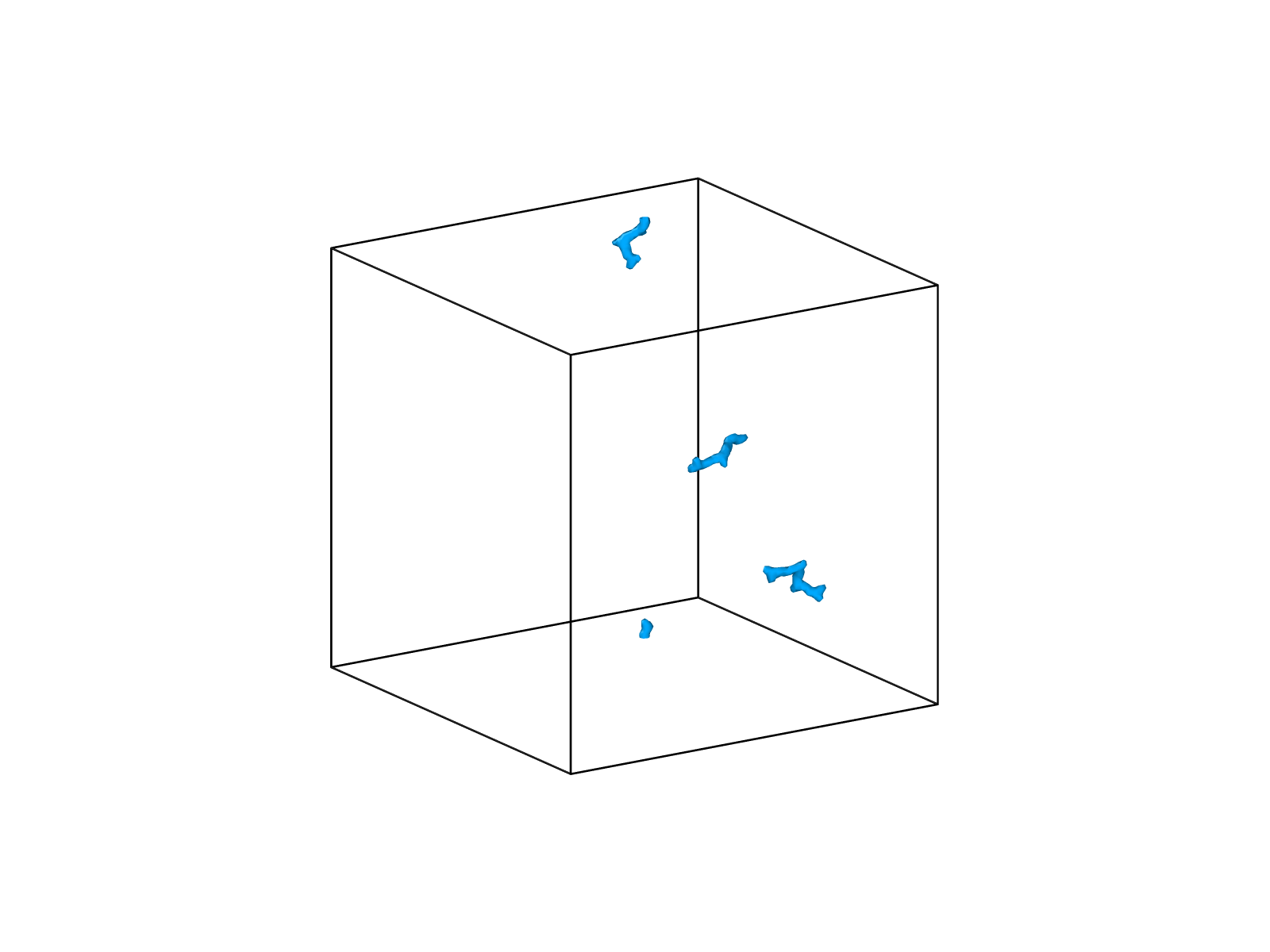

Supplement: Supplementary file 1 — ao4c07413_si_001.zip [file ao4c07413_si_001.zip › graphics/Results/amine_e50.png]

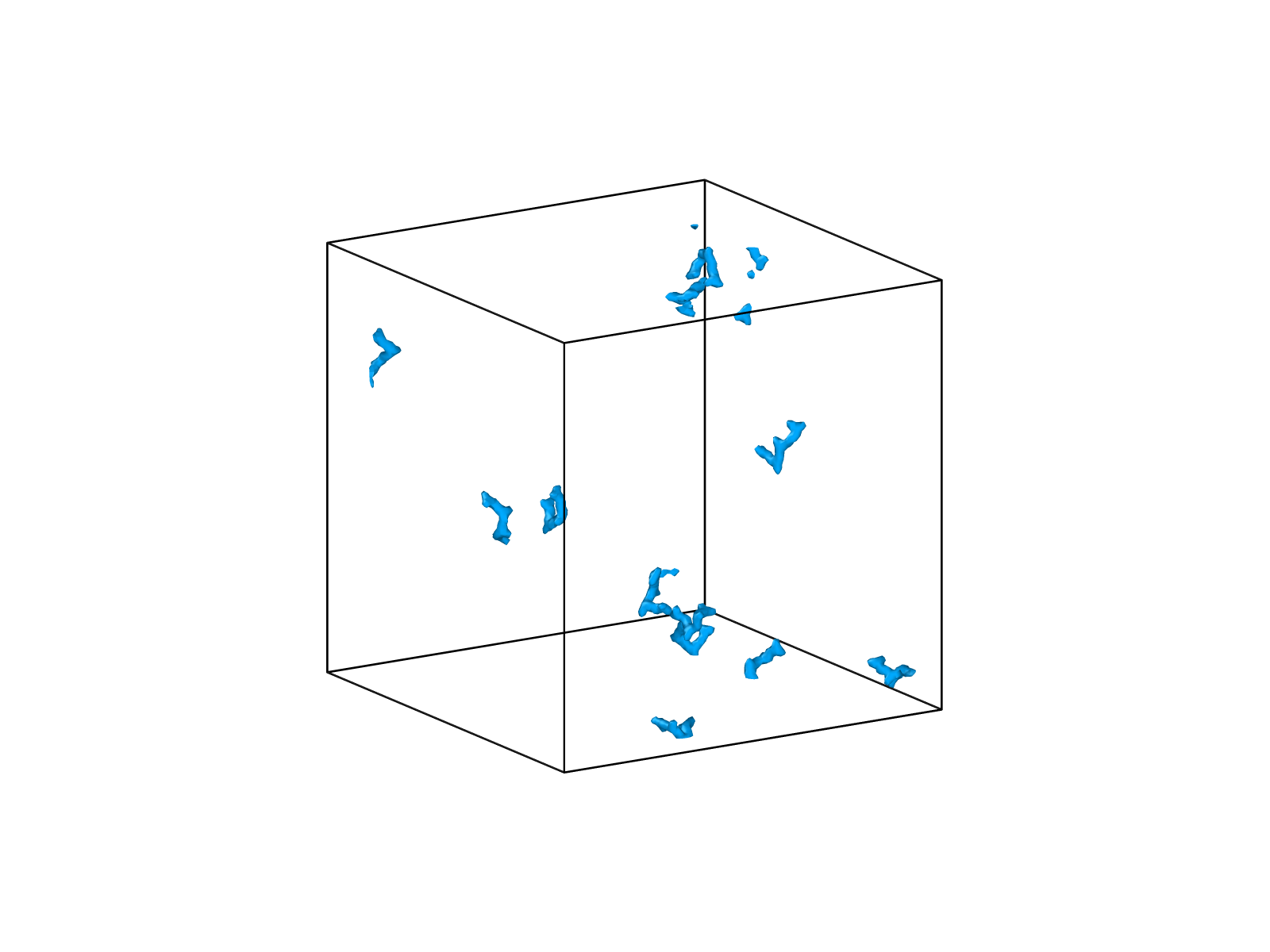

Supplement: Supplementary file 1 — ao4c07413_si_001.zip [file ao4c07413_si_001.zip › graphics/Results/amine_e75.png]

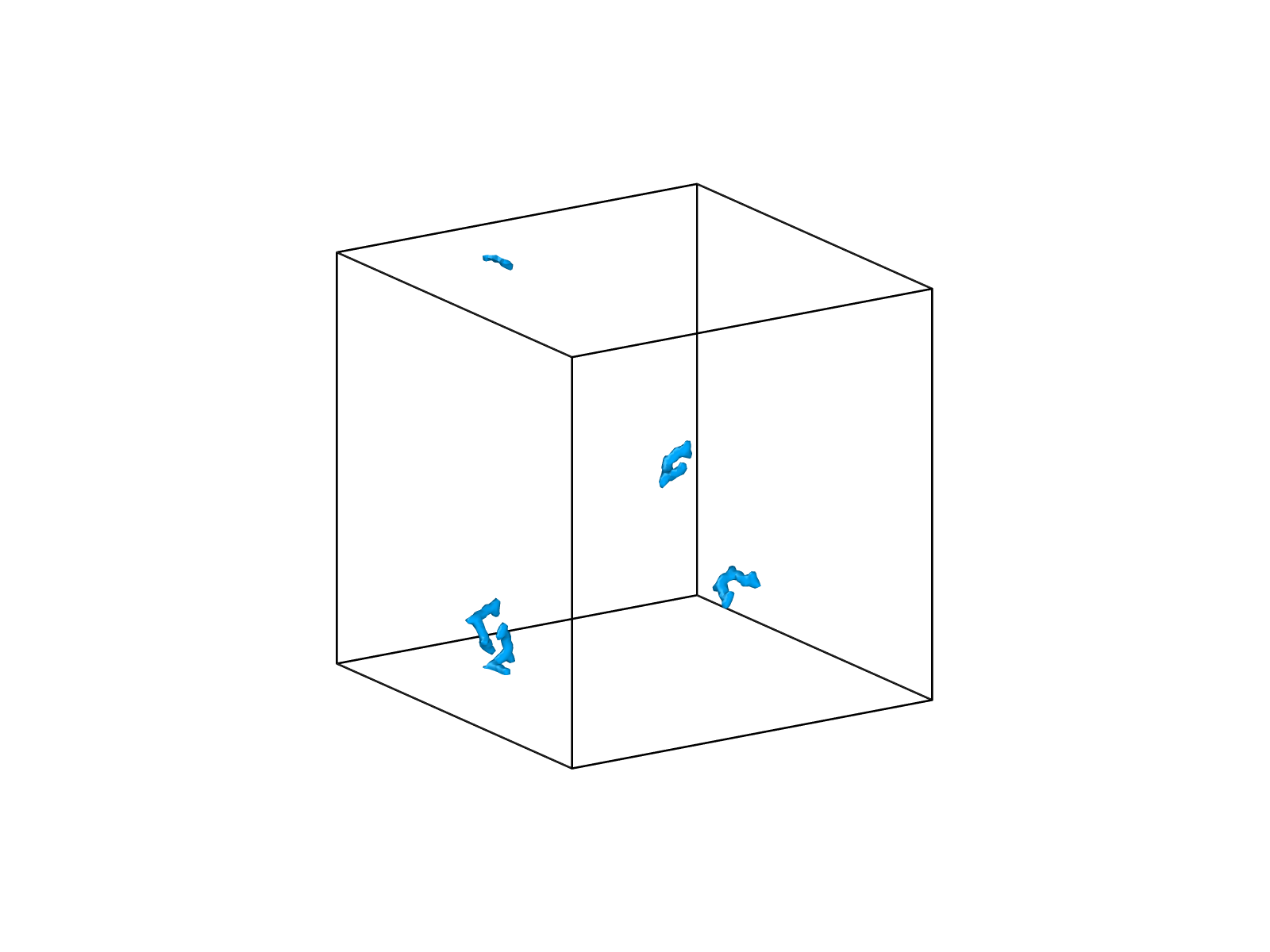

Supplement: Supplementary file 1 — ao4c07413_si_001.zip [file ao4c07413_si_001.zip › graphics/Results/amine_e375.png]

Glass Transition Temperature

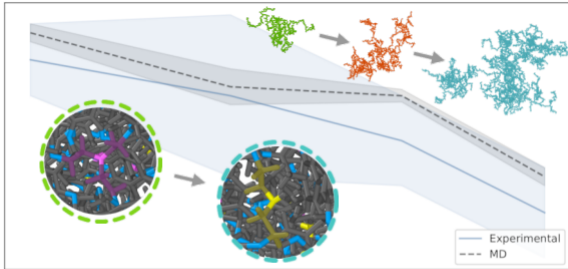

Amine Concentration

Supplement: Supplementary file 1 — ao4c07413_si_001.zip [file ao4c07413_si_001.zip › graphics/Results/graphical_abstracts_new6.pdf]

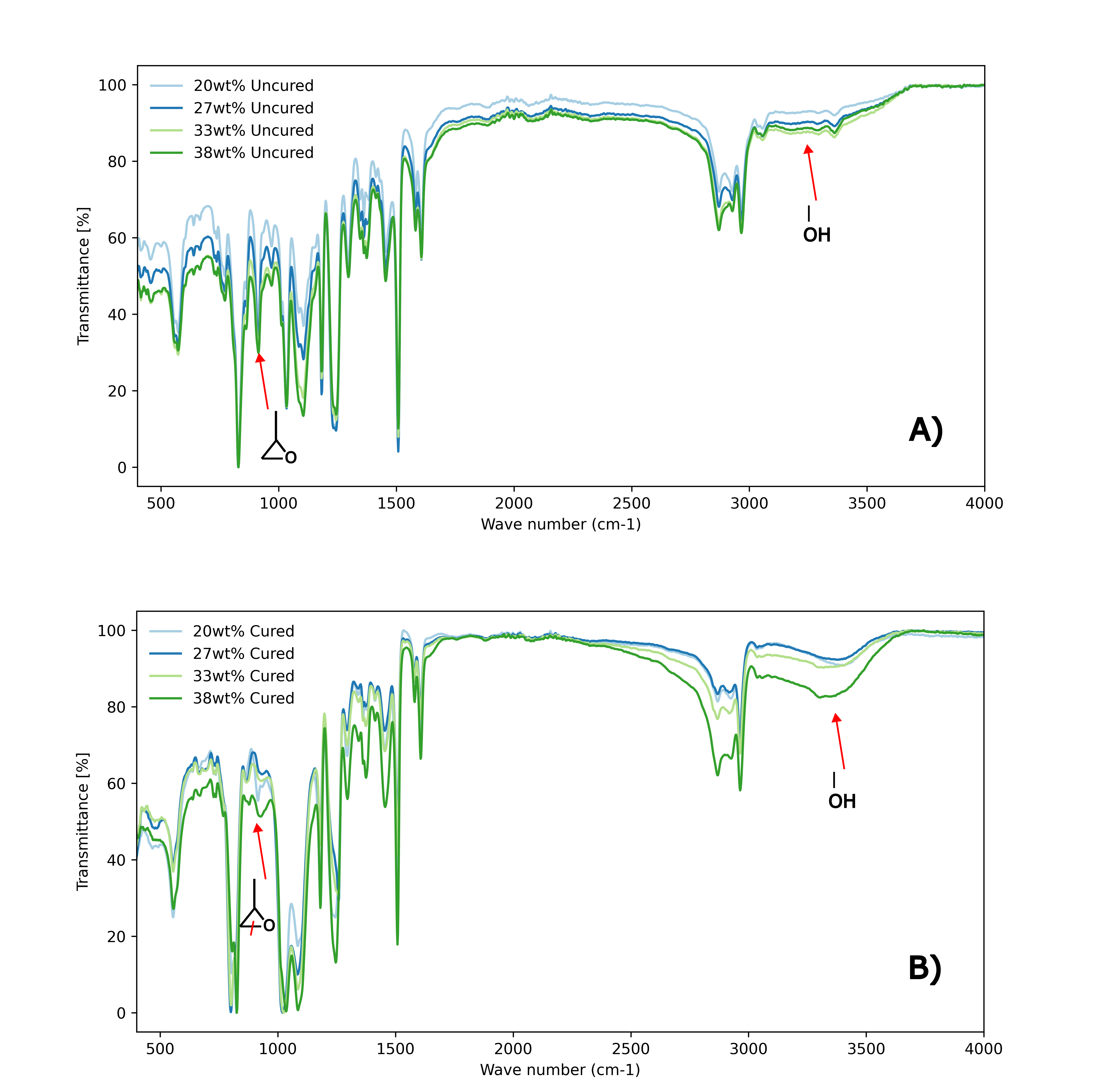

Supplement: Supplementary file 1 — ao4c07413_si_001.zip [file ao4c07413_si_001.zip › graphics/Results/FTIR wide.png]

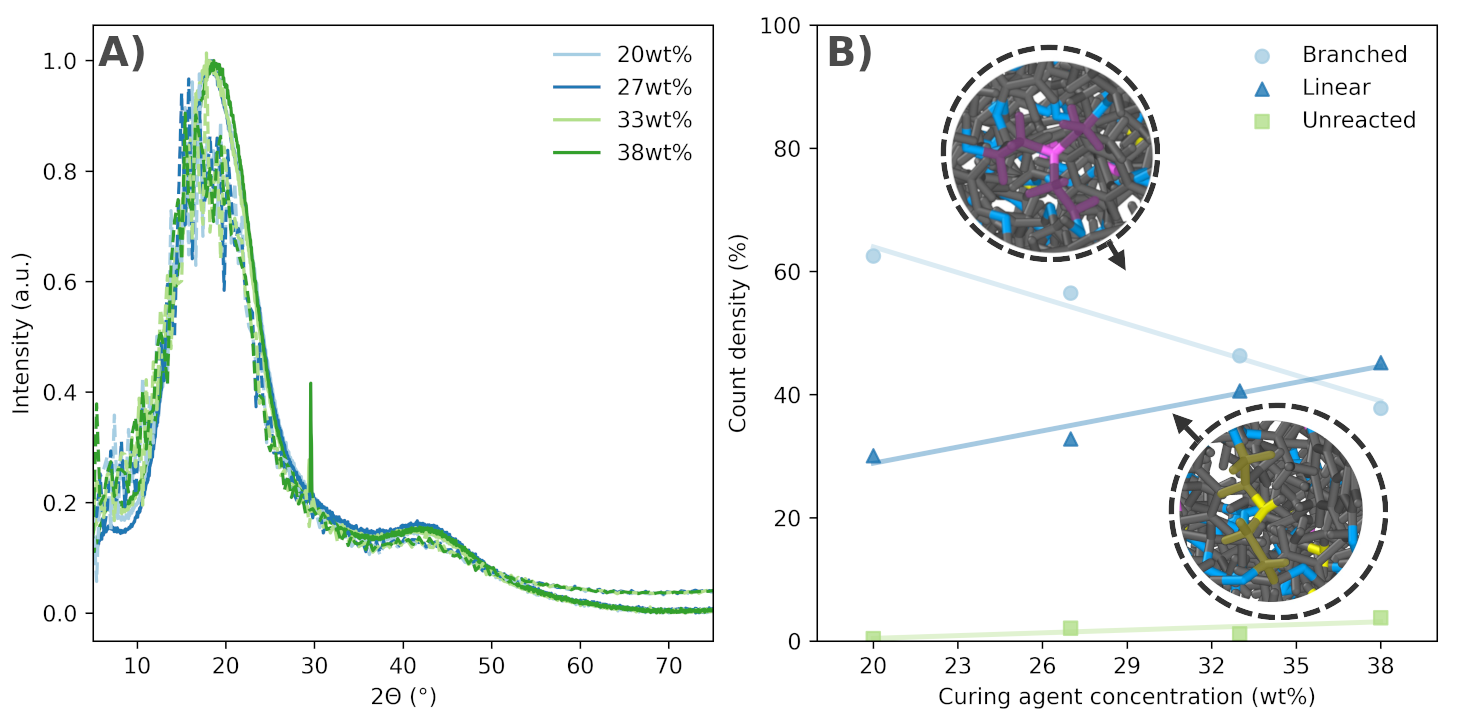

Supplement: Supplementary file 1 — ao4c07413_si_001.zip [file ao4c07413_si_001.zip › graphics/Results/xrd_count.png]

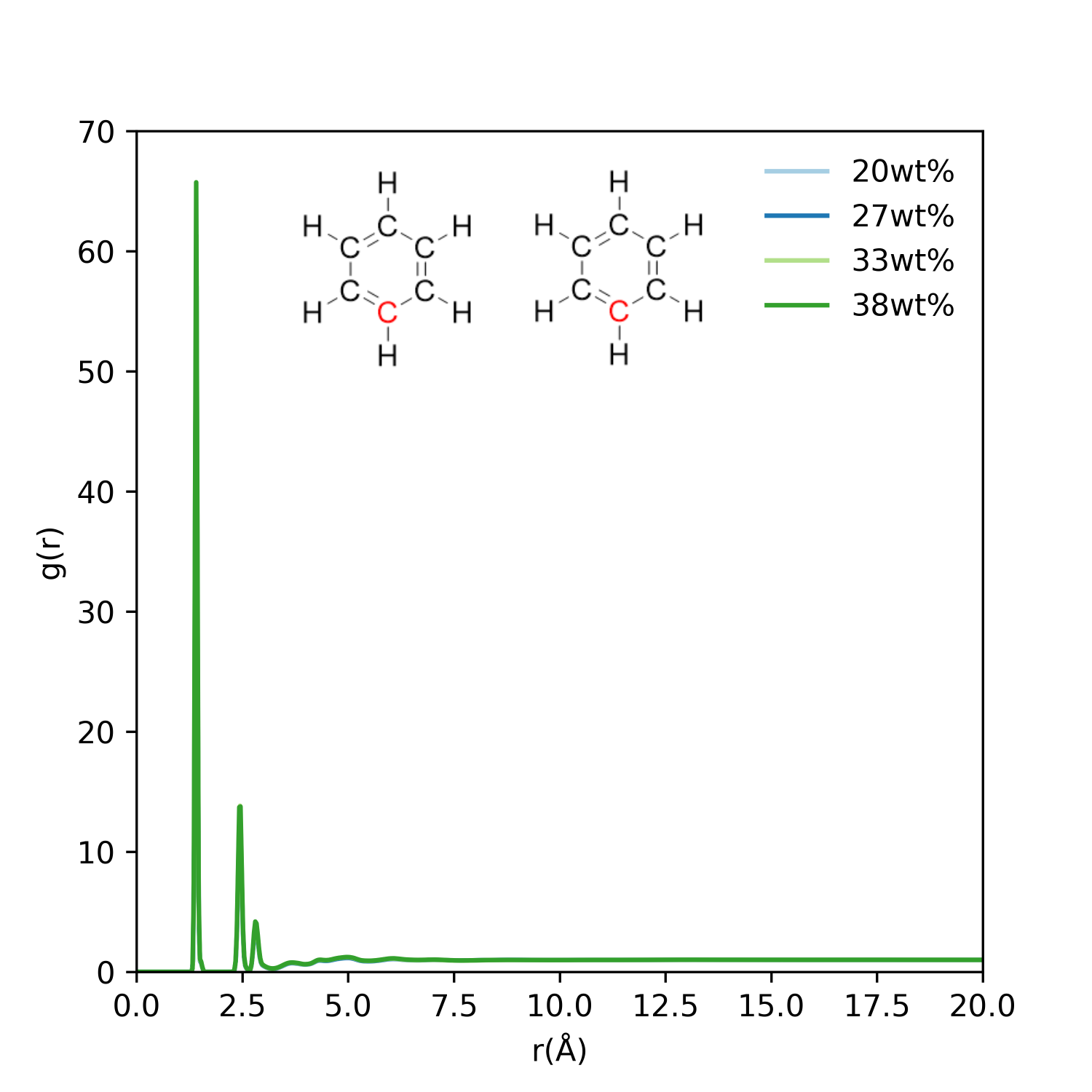

Supplement: Supplementary file 1 — ao4c07413_si_001.zip [file ao4c07413_si_001.zip › graphics/Results/RDF/Results rdf aromatic-aromatic supplementary.png]

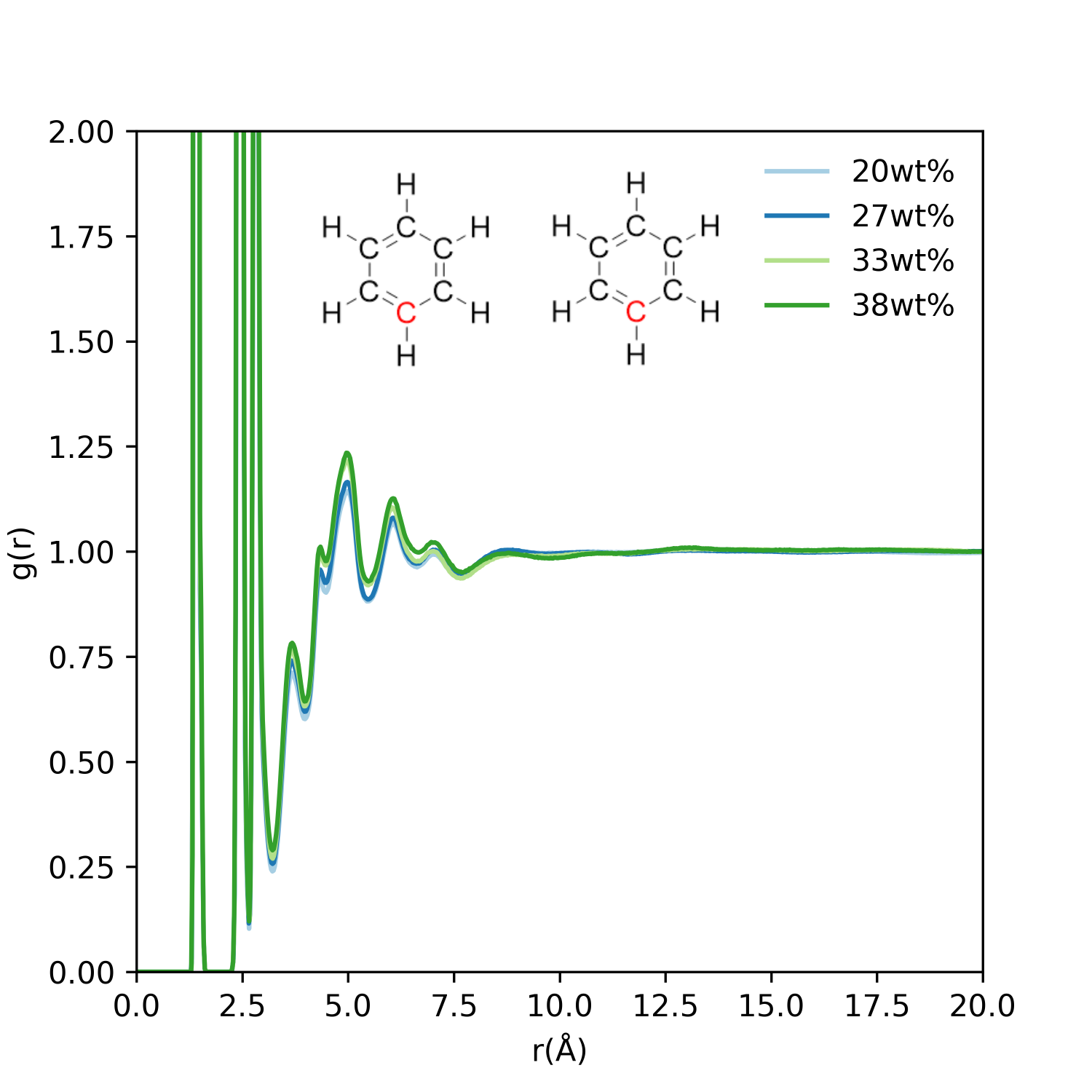

Supplement: Supplementary file 1 — ao4c07413_si_001.zip [file ao4c07413_si_001.zip › graphics/Results/RDF/Results rdf aromatic-aromatic.png]

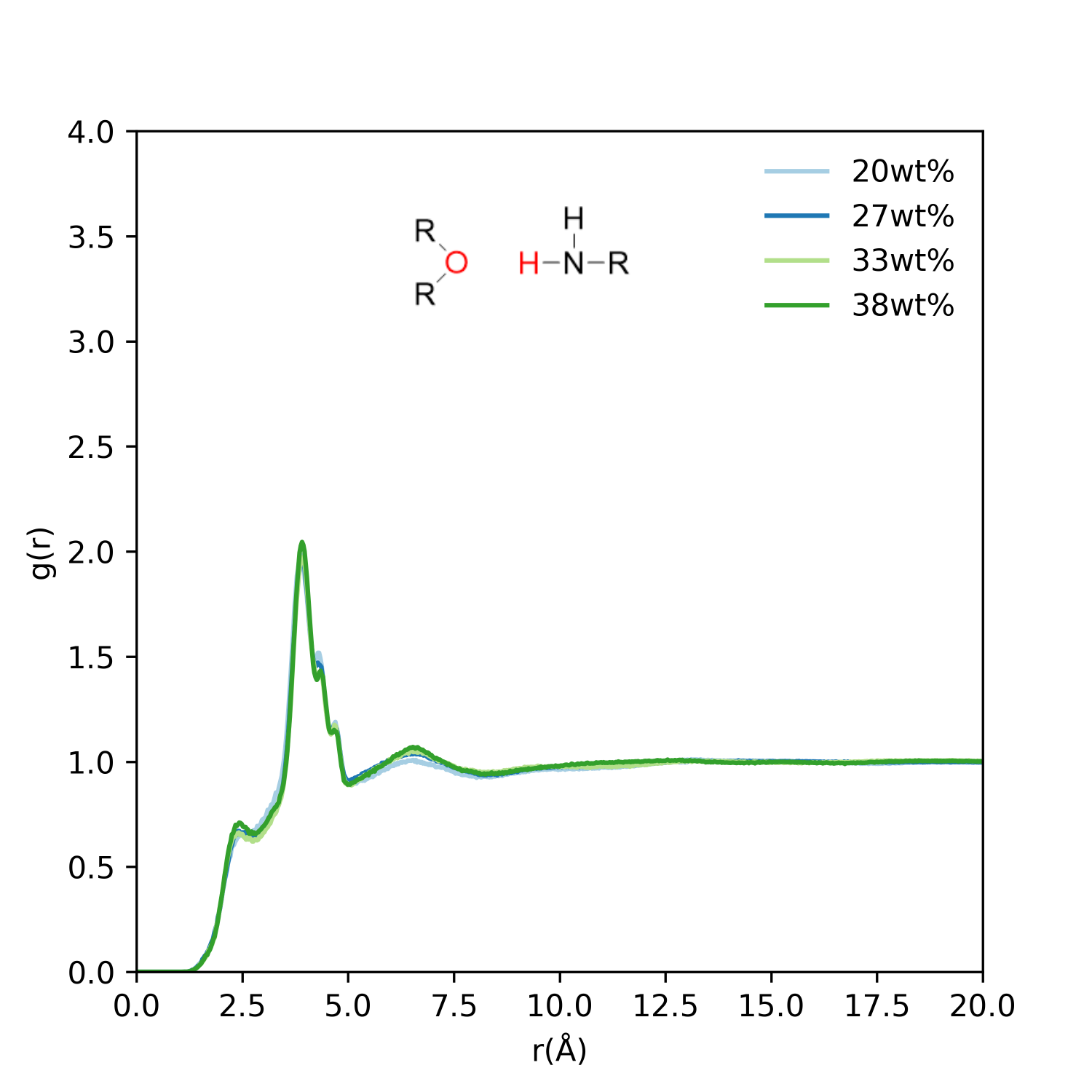

Supplement: Supplementary file 1 — ao4c07413_si_001.zip [file ao4c07413_si_001.zip › graphics/Results/RDF/Results rdf O-HN.png]
