# Supplementary material for: Effect of Variations of Amine Content and Network Branching on Thermomechanical Properties of Epoxy Systems
Source: ACS Omega. 2024 Dec 11;9(51):50414–26. doi: 10.1021/acsomega.4c07413 (PMC11683609; doi:10.1021/acsomega.4c07413)
Supplement: Supplementary file 2 — ao4c07413_si_002.pdf [file ao4c07413_si_002.pdf]

Supplementary Information

Effect of variations of amine content and network branching on thermomechanical properties of epoxy systems

Michael Robert Kelly,[*,†,‡], Arpenik Kroyan,[†,‡] Ingrid Hallsteinsen,[†] Sondre Kvalvåg Schnell,[†] Hilde Lea Lein[†]

†*Department of Materials Science and Engineering, Norwegian University of Science and Technology, NTNU, Sem Sælands vei 12, 7034 Trondheim, Norway*

‡ *These authors contributed equally*

E-mail: michael.r.kelly@ntnu.no

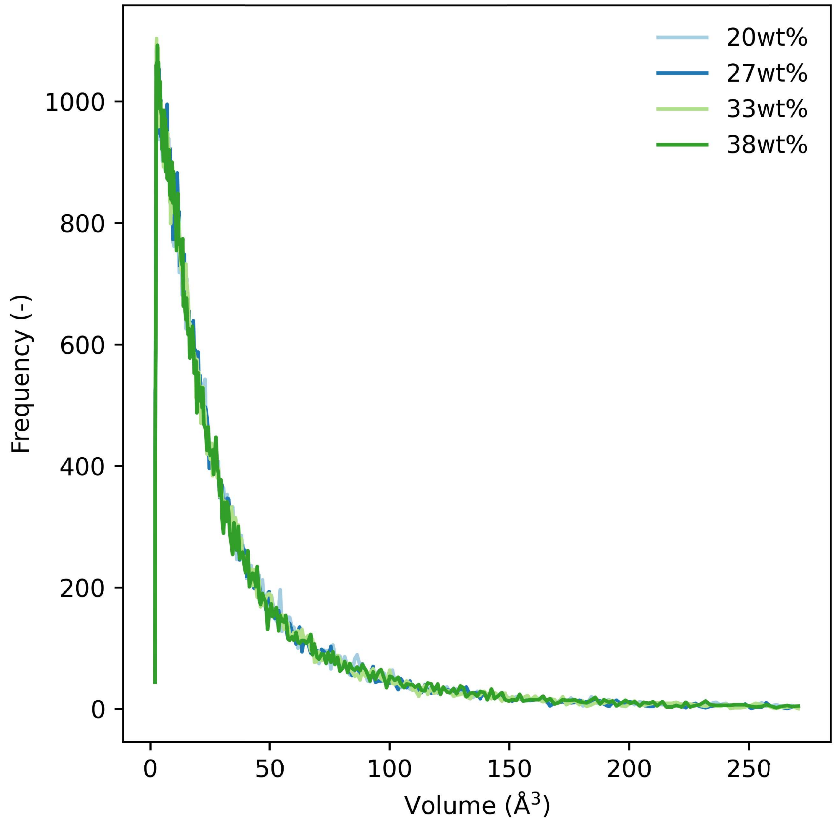

Figure S1: Probe particle volume distribution from simulations. From integration of the area under the curves we find the void volume of 6.4, 6.1, 5.8 and 5.5 % for the cured Epikote 828-Jeffamine D230 systems with 20wt%, 27wt%, 33wt% and 37wt% curing agent respectively, as acquired from molecular dynamics simulations.

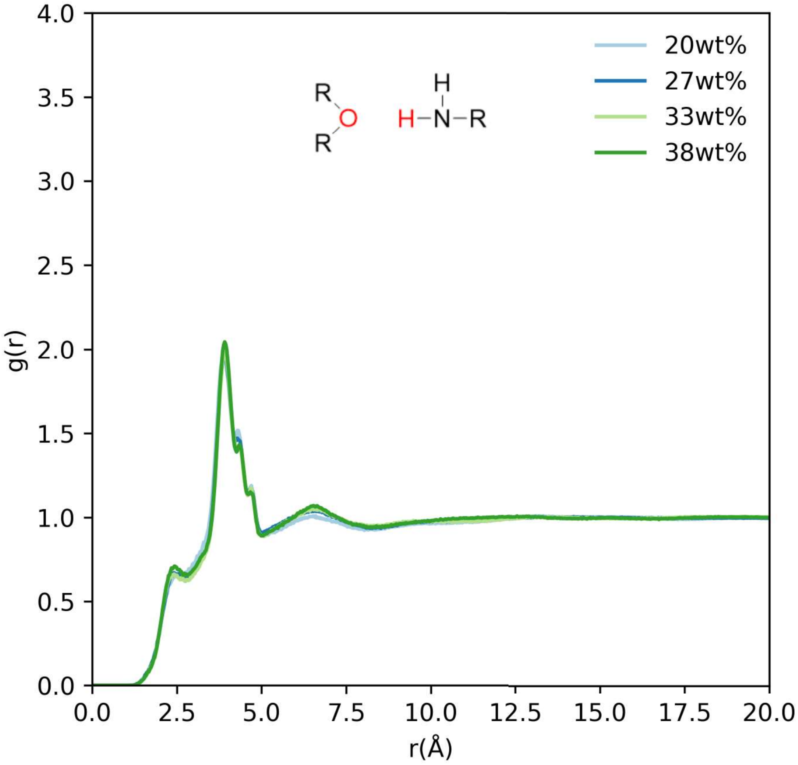

Figure S2: Radial distribution function of oxygen to amino hydrogen in the cured Epikote 828-Jeffamine D230 systems with varying curing agent concentrations, as acquired from molecular dynamics simulations.

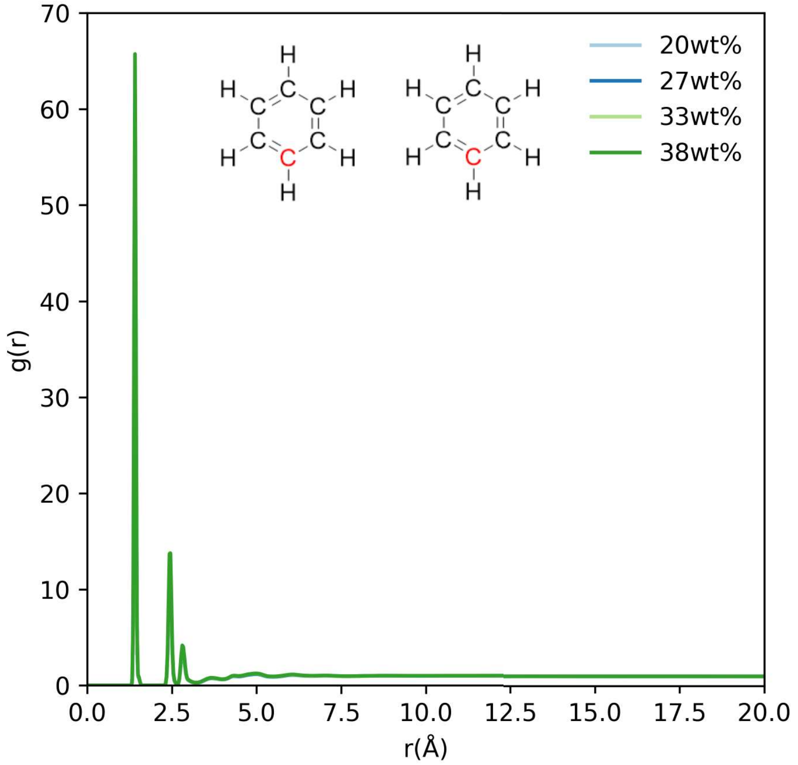

Figure S3: Radial distribution function of aromatic group to aromatic group in the cured Epikote 828-Jeffamine D230 systems with varying curing agent concentrations, as acquired from molecular dynamics simulations.

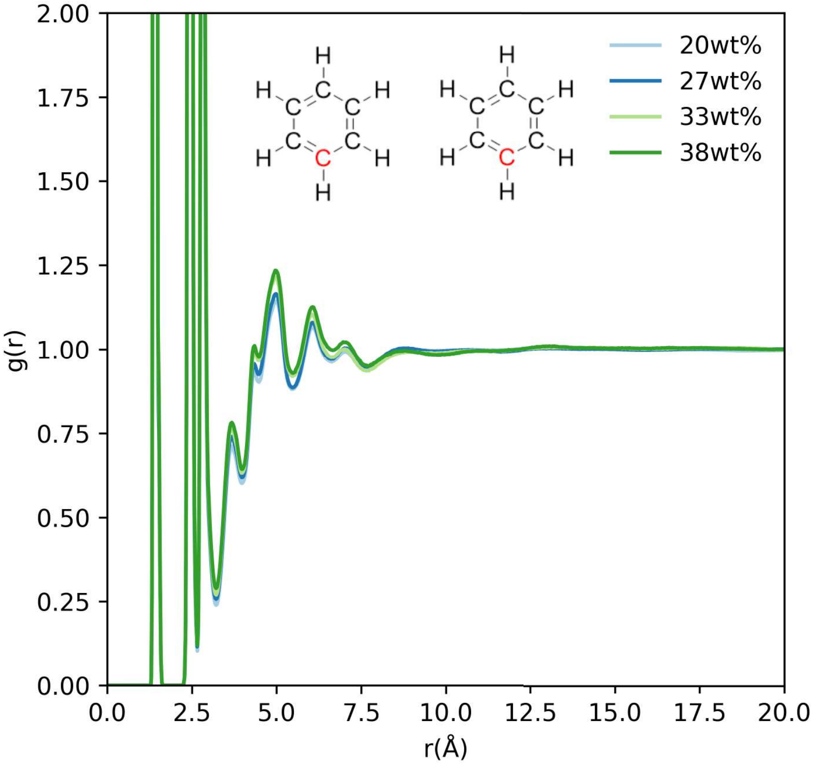

Figure S4: Region of interest of the radial distribution function of aromatic group to aromatic group in the cured Epikote 828-Jeffamine D230 systems with varying curing agent concentrations, as acquired from molecular dynamics simulations.

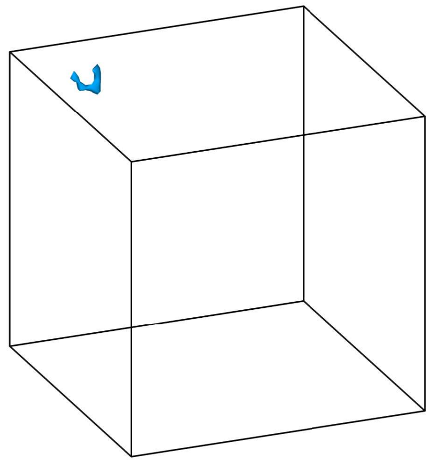

Figure S5: Snapshot of free amine distribution in the simulation box for system with 20 wt% curing agent (E20).

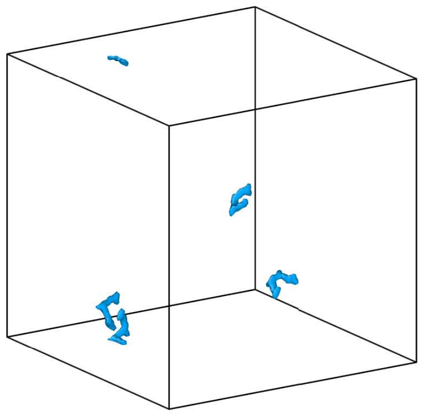

Figure S6: Snapshot of free amine distribution in the simulation box for system with 27 wt% curing agent (E27).

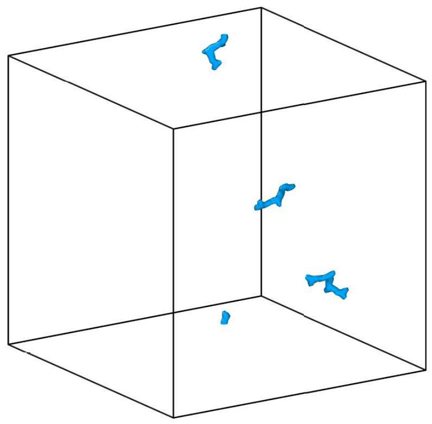

Figure S7: Snapshot of free amine distribution in the simulation box for system with 33 wt% curing agent (E33).

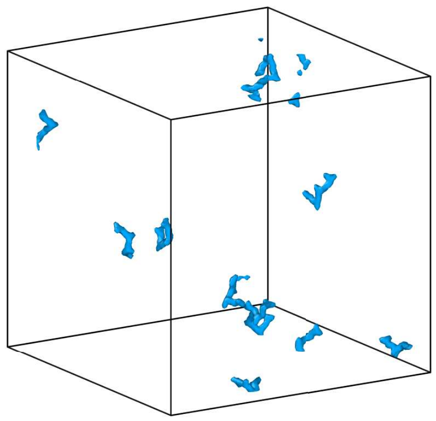

Figure S8: Snapshot of free amine distribution in the simulation box for system with 38 wt% curing agent (E38).
